# In silico analysis of embolism in cerebral arteries using fluid-structure interaction method

**DOI:** 10.1016/j.heliyon.2024.e30443

**Published:** 2024-04-27

**Authors:** Pouria Talebibarmi, Bahman Vahidi, Mahtab Ebad

**Affiliations:** Division of Biomedical Engineering, Department of Life Science Engineering, Faculty of New Sciences and Technologies, University of Tehran, Tehran, Iran

**Keywords:** Embolic stroke, Middle cerebral artery, Fluid-structure interaction, Visco-hyperelastic, Hyperelastic, Non-Newtonian fluid

## Abstract

Ischemic stroke, particularly embolic stroke, stands as a significant global contributor to mortality and long-term disabilities. This paper presents a comprehensive simulation of emboli motion through the middle cerebral artery (MCA), a prevalent site for embolic stroke. Our patient-specific computational model integrates major branches of the middle cerebral artery reconstructed from magnetic resonance angiography images, pulsatile flow dynamics, and emboli of varying geometries, sizes, and material properties. The fluid-structure interactions method is employed to simulate deformable emboli motion through the middle cerebral artery, allowing observation of hemodynamic changes in artery branches upon embolus entry. We investigated the impact of embolus presence on shear stress magnitude on artery walls, analyzed the effects of embolus material properties and geometries on embolus trajectory and motion dynamics within the middle cerebral artery. Additionally, we evaluated the non-Newtonian behavior of blood, comparing it with Newtonian blood behavior. Our findings highlight that embolus geometry significantly influences trajectory, motion patterns, and hemodynamics within middle cerebral artery branches. Emboli with visco-hyperelastic material properties experienced higher stresses upon collision with artery walls compared to those with hyperelastic properties. Furthermore, considering blood as a non-Newtonian fluid had notable effects on emboli stresses and trajectories within the artery, particularly during collisions. Notably, the maximum von Mises stress experienced in our study was 21.83 kPa, suggesting a very low probability of emboli breaking during movement, impact, and after coming to a stop. However, in certain situations, the magnitude of shear stress on them exceeded 1 kPa, increasing the likelihood of cracking and disintegration. These results serve as an initial step in anticipating critical clinical conditions arising from arterial embolism in the middle cerebral artery. They provide insights into the biomechanical parameters influencing embolism, contributing to improved clinical decision-making for stroke management.

## 1 Introduction

Ischemic stroke, constituting about 87 % of all strokes in the United States [1], occurs when blood vessels supplying the brain are blocked. This blockage leads to an inadequate supply of blood, depriving brain cells of essential nutrients such as glucose and oxygen. Consequently, this deprivation results in the eventual death of brain tissue [2]. Most evidence suggests that the majority of ischemic strokes are embolic strokes in nature [3], where a blood clot forms in another part of the body and travels to the brain and typically block the anterior, middle or posterior cerebral artery, or one of their branches. Studying this phenomenon is crucial for gaining a better understanding of the underlying mechanisms and risk factors associated with ischemic strokes. Such research can contribute to the development of more effective preventive strategies, early detection methods, and targeted treatments, ultimately improving outcomes for individuals at risk of or affected by ischemic strokes. Researchers actively pursue this understanding by employing various investigative approaches, including the use of animal models, in vitro studies, and computational analyses.

In recent years, the employment of animal models has significantly contributed to enhancing our comprehension of the pathophysiological mechanisms underlying strokes [[4], [5], [6]]. Various animal species have been utilized in stroke studies; however, concerns exist regarding the accuracy and precision of these investigations [7].

In an in vitro embolic stroke model conducted by Ref. [8], the movement and formation of emboli within the circle of Willis were explored. Clots used in their study were derived from the blood of a crab species with similarities to human blood, and the vessel model was generated from magnetic resonance imaging. Their notable research achievements encompassed the analysis of hemodynamic parameters in blood flow, embolus movement trajectories, the distribution ratio of emboli in cerebral vessels, and the formation process of clots. Another study by Ref. [9] demonstrated that smaller emboli with irregular shapes, specifically thrombus fragments, have the potential to induce intracranial infarction. Additionally, it was noted that the extent of damage caused by embolism depends not only on its size but also on the type of compounds involved and the shape of the embolus. Chung's study focused on investigating the influence of parameters such as fluid flow volume, arterial morphology, and clot size on the trajectory of clots within cerebral arteries [10].

Given the considerable time and financial investments involved in laboratory methods, along with the challenges of measuring specific parameters, numerical simulations provide an effective way to overcome these obstacles and explore the desired variables [[11], [12], [13], [14], [15], [16], [17]]. Computational simulation of blood vessels proves to be a powerful tool for investigating the environmental behavior of the circulatory system relevant to this disease, all in a completely non-invasive manner.

In the study conducted by Ref. [18], the trajectories of embolic particles released from the carotid artery and basilar artery within the cerebral artery networks were investigated. Moreover, they simulated the impact of particle size and density on path selection using numerical modeling. In the study by Ref. [19], the role of the anatomy of the Circle of Willis in stroke was explored. The results demonstrated that anatomical changes in the Circle of Willis significantly influenced the distribution of emboli in the six major cerebral arteries. In the study conducted by Ref. [20], the geometry of the Circle of Willis was generated using medical images of a patient's cerebral vascular anatomy. Subsequently, a fluid-structure interaction computational algorithm was employed to investigate and analyze the influence of blood clot flexibility on their movement dynamics within the vascular network. The results of their research indicate that mechanical parameters play a pivotal role in determining the trajectory of blood clots, with stiffer clots exhibiting a greater tendency to enter the larger branches of the brain. In another study by Ref. [21], the motion of clots inside a branch of pulmonary arteries, and the effect of their mechanical properties on their movement and the stress they experienced, was investigated. Another study revealed that employing 3D Monte Carlo simulations for embolic stroke enables predictions regarding the sizes and locations of lesions resulting from particular embolization patterns [22].

In silico studies offer promising avenues for advancing our comprehension of acute ischemic stroke procedures, facilitating further optimization of devices and procedures, and enhancing clinical training methodologies [23]. Additionally, patient-specific investigations hold potential for predictive analyses of future stroke occurrences and non-invasive assessment of stroke risk in individual patients [24].

In this study, we employed magnetic resonance angiography images to create an image-based geometry of the MCA, allowing us to simulate the embolic stroke phenomenon from a biomechanical perspective. In contrast to many previous studies that assumed emboli to have a spherical geometry, an idealized assumption not reflective of real conditions, we considered various geometries with different sizes for the emboli in our investigation. Additionally, we explored different material properties and release times inside the artery. Furthermore, we delved into the effects of blood flow on the artery wall in the presence of emboli, examining alterations in blood velocity within the branches. Unlike previous studies that often treated blood as a Newtonian fluid, we conducted analyses considering the non-Newtonian behavior of blood. Comparisons with the Newtonian fluid assumption revealed significant differences in the outcomes, providing a more nuanced understanding of the fluid dynamics in the presence of emboli.

## 2 Material and methods

### 2.1 Geometry construction

The MCA has been selected as the focal point of our study. This decision is driven by two primary reasons. Firstly, the MCA is a prevalent site for ischemic stroke and artery occlusion, making it a critical area for investigation. Secondly, occlusion in the MCA can result in severe complications, including paralysis of the opposite limb, speech impairment, loss of consciousness, etc [25].

**Fig. 1:**
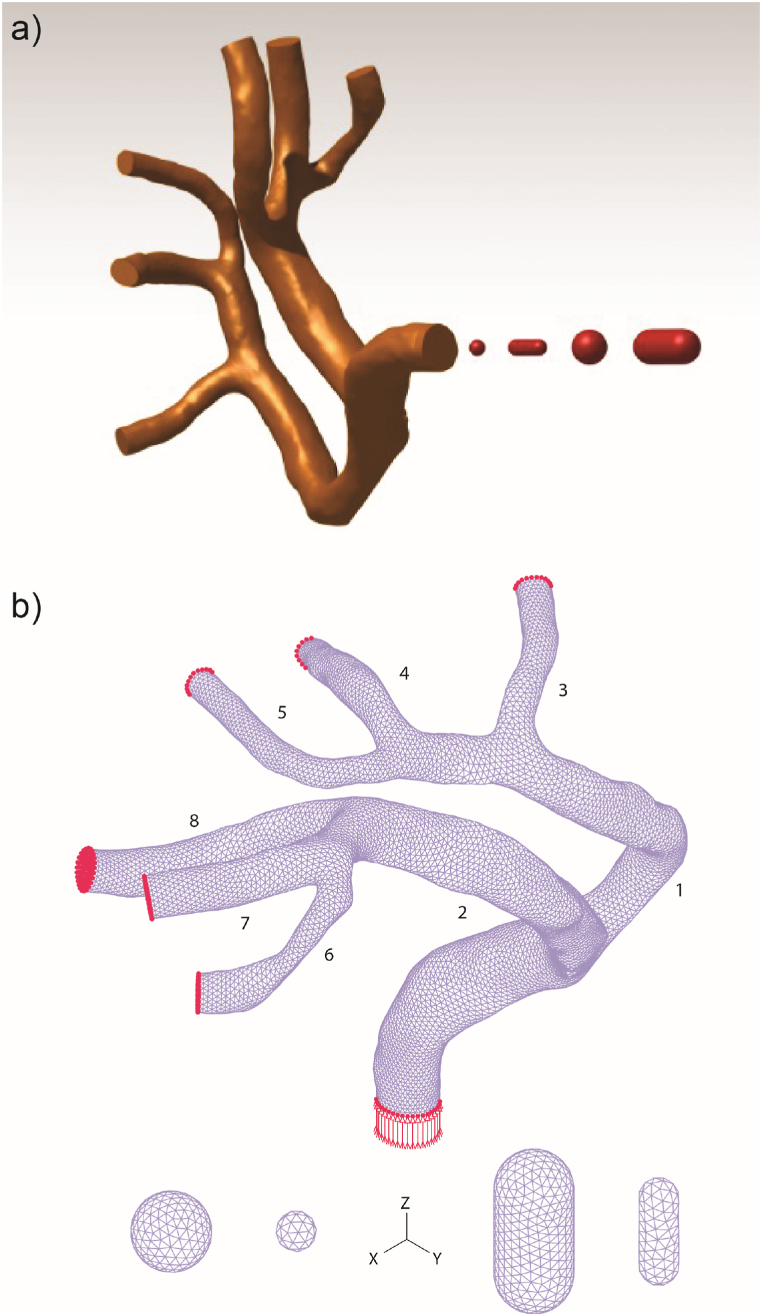
a) Crafted 3D geometry of the MCA and the emboli, b) Discretized geometry of the MCA and the emboli (the numbers depict the branch numbers).

To construct the desired geometry, we utilized magnetic resonance angiography (MRA) images of the healthy MCA from a patient in digital format. These images comprised 176 individual frames, which were employed to generate point clouds from the model using Mimics Innovation Suite software (Materialise Mimics Innovation Suite Medical. v19.0). Once the desired region was identified, the resulting point cloud was imported into CATIA software, where the initial geometry model was crafted (as shown in Fig. 1a). The diameter of the main branch of the MCA obtained in our research aligns closely with the physiological range, affirming the accuracy of our findings (Table 1).

**Table 1:** Comparison of main branch diameter of MCA obtained in this investigation with that of previous studies.

Artery	MCA main branch diameter (mm)			
	Our study	[26]	[27]	[28]
MCA	3	3	2.86	3.1

### 2.2 Properties of embolus

A Mooney-Rivlin model, specifically designed for rubber/foam materials, was utilized to simulate the elastic response of the clot. The stress-strain curve, derived by fitting experimental data from Ref. [29] into the Mooney-Rivlin curve, was incorporated into ADINA software (refer to the supplementary materials for the corresponding plot, Fig. S1). Additionally, to explore the impact of viscoelasticity on hemodynamics and embolus behavior, a Visco-hyperelastic model was employed for the embolus. The viscoelastic coefficients were obtained from the generalized Maxwell model, a model tailored for viscoelastic materials akin to living tissues [30]. Equation (3) describes the stress in terms of viscoelastic constants, as outlined in Eqs. (1) and (2) [21].

(1) $$\tau^{\alpha }=\frac{\eta^{\alpha }}{E^{\alpha }}$$

(2) $$\beta^{\alpha }=\frac{E^{\alpha }}{E^{\infty }}$$

(3) $$\sigma =\int_{0}^{1}E^{\infty }\left[1+\sum_{\alpha }\beta^{\alpha }\;\exp\;\left(-\frac{t-\overset{´}{t}}{\tau^{\alpha }}\right)\right]\dot{e\;}d\overset{´}{t}$$

In the equations presented above, t represents time, τ denotes relaxation time, β stands for viscoelastic factor, η represents viscosity, E signifies elasticity, and σ corresponds to stress in the generalized Maxwell model. Referencing the discoveries outlined in Ref. [30] and the aforementioned associations, the viscoelastic coefficients are detailed in (Table 2) as follows:

**Table 2:** Mechanical properties of the emboli [30].

$E^{\infty }\left(Pa\right)$	$E^{1}\left(Pa\right)$	$E^{2}\left(Pa\right)$	$\eta^{1}\left(Pa.s\right)$	$\eta^{2}\left(Pa.s\right)$	$\tau^{1}\left(s\right)$	$\tau^{2}\left(s\right)$	$\beta^{1}$	$\beta^{2}$
518.42	1.3 × 10^15^	748.66	0.13	2.95	10^–16^	0.00394	25 × 10^11^	1.444

According to previous studies, the density of the clot is equal to 1050 kg/m³ [10,18,31]. Marder et al.’s investigation [32] provided details on the size and dimensions of clots obtained from the MCA and internal carotid artery. In our study, we opted for four sizes from the reported values in the MCA and represented them in both spherical and elliptical shapes, as outlined in Table 3. To enhance comparability, clots with substantial differences in size were chosen. Additionally, to highlight the impact of elliptical and spherical geometry, clots were categorized into two groups with identical width but varying lengths.

**Table 3:** Geometry models and dimensions of the emboli.

Model	Geometry	Dimension (mm)
$S_{1}$	spherical	Diameter = 1
$E_{1}$	elliptical	Width = 1 & Length = 3
$S_{2}$	spherical	Diameter = 2.2
$E_{2}$	elliptical	Width = 2.2 & Length = 5

### 2.3 Properties of blood

In our investigation, we have considered blood flow to be laminar and incompressible, maintaining a density of 1050 kg/m³ [20,33]. It's important to note that blood exhibits non-Newtonian behavior, but it's also an acceptable assumption to treat it as a Newtonian fluid when the shear rate exceeds 100 s⁻^1^ [34]. Previous research has supported this assumption for the MCA [35,36]. However, some studies emphasized the importance of considering the non-Newtonian characteristics of blood in situations involving artery abnormalities like aneurysms [[37], [38], [39]]. Furthermore, bifurcations, which are prevalent in our geometry, are crucial locations to evaluate the influence of blood's non-Newtonian behavior [40]. Given that our geometry contains multiple bifurcations and considering that the presence of a clot in the artery could significantly impact blood flow, we decided to investigate the role of blood's non-Newtonian behavior in our simulations.

To account for this, alongside treating blood as a Newtonian fluid with a viscosity of 3.48 cP [20], we employed the Carreau model, which accurately captures the shear-thinning behavior of blood [41]. In this model, the fluid's viscosity changes according to the following equations [41]:

(4) $$\mu =\mu_{\infty }+\left(\mu_{0}-\mu_{\infty }\right)\cdot {\left(1+K\dot{\gamma_{ij}^{2}}\right)}^{n}$$

(5) $${\dot{\gamma }}_{ij}=\left(\frac{\partial \upsilon_{i}}{\partial x_{j}}+\frac{\partial \upsilon_{j}}{\partial x_{i}}\right)$$

Where ${\dot{\gamma }}_{ij}$ is shear rate, K and n are constant coefficients, $\mu_{\infty }$ and $\mu_{0}$ are viscosities at infinite and zero shear rate, respectively (see Table 4).

**Table 4:** Non-Newtonian model of blood [41].

μ∞(N.sm2)	μ0(N.sm2)	K	n
0.00345	0.056	10.976	−0.3261

### 2.4 Boundary conditions

**Fig. 2:**
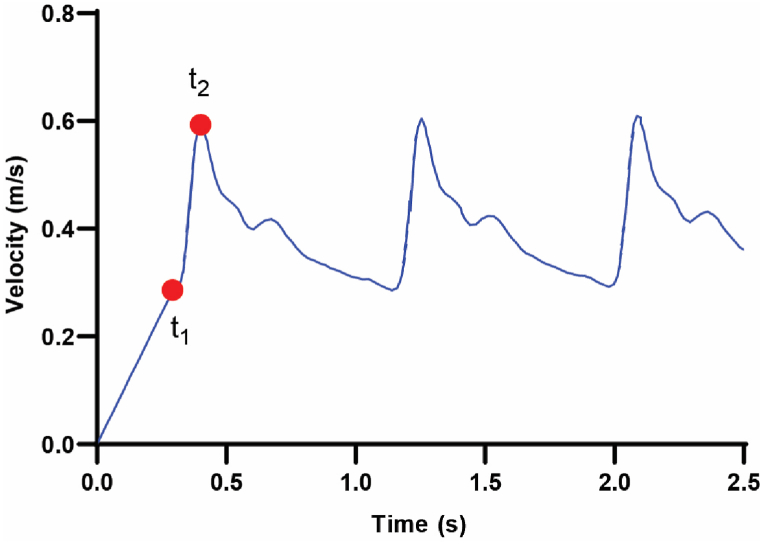
Blood flow velocity waveform and emboli release times.

Assigning the FSI boundary condition to the surface of the emboli, we also treated the wall of the artery as the wall boundary, imposing a No-slip condition. The blood flow velocity waveform in the MCA under normal conditions was extracted from a transcranial Doppler ultrasound study conducted by Ref. [42], as illustrated in (Fig. 2) and the pressure on the outlet boundaries was set to zero. To explore the impact of release time on the clot, it was introduced into the artery at two distinct moments — either at the initiation of systole or at the end of systole. The corresponding release times are depicted in Fig. 2.

Moreover, we set the friction coefficient between the artery wall and the clot to 0.3 [43]. In order to establish a well-developed flow condition, we considered development lengths of 47 mm at the inlet and 28 mm at the outlet [44].

### 2.5 Numerical domains and governing equations

The generation of tetrahedral mesh for both fluid and solid domains was accomplished using Hypermesh (Hypermesh, Altair Hyperworks) software. The governing equations for this phenomenon involve the conservation of mass and momentum, addressed through the arbitrary Lagrangian-Eulerian (ALE) formulation for the fluid. In FSI solutions where significant structural interfacial displacement occurs, the ALE formulation facilitates the reliable movement of the mesh network during the solution by concurrently employing pure Eulerian and Lagrangian methods [45]. The motion of the clot was governed by the General Lagrangian formulation [46]. A brief summary of the conservation of mass and momentum in ALE form is provided below (Eqs. (6)–(8)):

(6) $${\frac{\partial \rho }{\partial t}|}_{\chi }+c\cdot \nabla \rho =-\rho \nabla \cdot \upsilon$$

(7) $$\rho \left({\frac{\partial \upsilon }{\partial t}|}_{\chi }+\left(c\cdot \nabla \right)\upsilon \right)=\nabla \cdot \sigma +\rho b$$

(8) $$c=\upsilon -\hat{\upsilon }$$

In the provided equations, ${|}_{\chi }$ denotes "holding the referential coordinate χ fixed." Here, ρ represents density, υ stands for material velocity, b is the specific body force, σ denotes the Cauchy stress tensor, and $\hat{\upsilon }$ represents mesh velocity [45]. Additionally, the displacement compatibility (kinematic condition) and dynamic condition, applied to the FSI boundaries, are expressed as follows (Eqs. 9 and 10) [47]:

(9) $$d_{f}=d_{s}$$

(10) $${n\cdot \tau }_{f}={n\cdot \tau }_{s}$$

Where, $d_{f}$ and $d_{s\;}$ represent the displacements of the fluid and solid, respectively, while $\tau_{f}$ and $\tau_{s}$ denote the stress of the fluid and solid, respectively. The vector "n" signifies the normal unit vector of the interface, with its values computed at the shared boundary between the fluid and solid.

### 2.6 Solution method

**Fig. 3:**
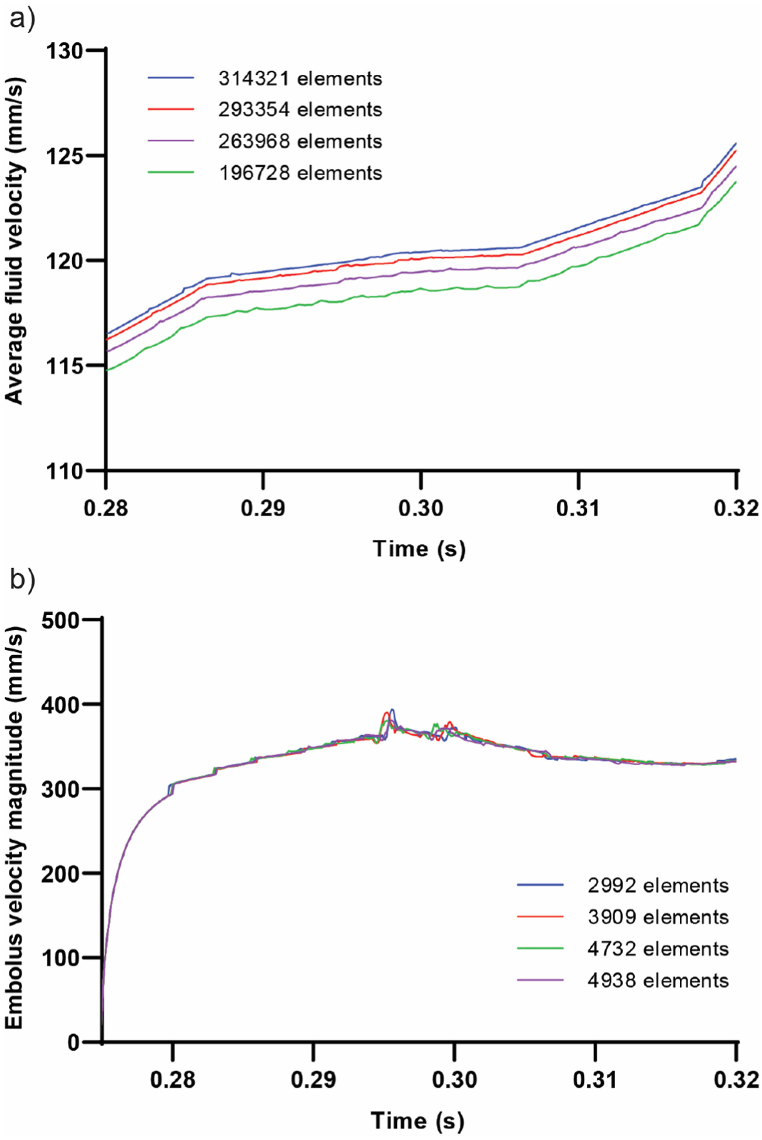
Mesh independence Analysis: a) Mesh size effects on average fluid velocity in branch number 8. b) Mesh size effects on embolus velocity within the MCA (Note: Panel b depicts the mesh for the embolus, while panel a represents the mesh for the fluid domain.).

Applying an FSI module, the coupled fluid and structure model underwent resolution using a finite element package (ADINA, Adina R&D Inc., Watertown MA, USA). The optimal approach for tackling this issue involved employing a sparse solver based on Gauss elimination and direct computational two-way coupling, proven to be swifter and more precise than the iterative coupling method [48]. In the direct coupling method, fluid and solid equations were simultaneously addressed within a unified system. A relative pressure tolerance of 0.001 was employed, with the displacement and stress relaxation factor set at 0.8. Tailored to the embolus's location in the artery, distinct time steps were implemented, featuring a maximum of 0.01 s and a minimum of 6 × 10^−5^ s. The iteration number for each solution time adhered to 500 in the Newton-Raphson scheme. Recognizing the intricate nature of embolus movement through the artery and the critical need to prevent mesh distortion during simulation, a steered adaptive mesh modification process was executed [49]. This process ensured that, in the event of mesh network disruption due to clot motion, it would be restored to sustain the solution procedure. Additionally, to model contact between the rigid artery wall and embolus, the artery's wall surface served as the target surface, while the clot's external surface acted as the contactor surface. To facilitate the steered adaptive mesh modification process, it was imperative to define an offset between contact surfaces; in this study, the offset was set at 0.1 mm. Furthermore, a compliance factor of 0.0001 was employed. To examine the solution's grid size independence within the fluid domain, we constructed four models, each with a distinct number of elements. It's noteworthy that the embolus ($E_{2}$), remained consistent across all four models. The fluid velocity in the middle region of the branch number 8 (Fig. 1b) was calculated (shown in Fig. 3a). To investigate mesh independence within the solid domain, we conducted a study employing varying numbers of elements for the embolus ($E_{2}$), and its velocity was evaluated as illustrated in (Fig. 3b), and the most appropriate size of elements for solid and fluid domains were selected.

## 3 Results and discussion

The primary objective of this project was to employ precise engineering software to simulate the occurrence of thromboembolism. Specifically, we aimed to model the movement of a blood clot within the MCA and its interaction with the surrounding blood. Through this simulation, we sought to examine the influence of various mechanical parameters on the hemodynamic characteristics of the blood flow and the morphological changes in the clot.

Validating the accuracy of the boundary conditions, we assessed the pressure distribution in the MCA and the blood flow rate in the main branch. The obtained results indicated that both parameters fell within the physiological range, as illustrated in Fig. S2 and Table 5.

**Table 5:** Comparison of blood flow in the main branch of MCA: Current investigation vs. previous studies.

Artery	Blood flow (ml/s)			
	Our study	[20]	[27]	[26]
MCA	2.5	2.55	2.78	2.44

### 3.1 Embolus motion dynamics and shear stress analysis

Hemodynamic factors, including shear stress generated by blood flow, play a pivotal role in regulating biochemical reactions such as the formation, growth, and lysis of clots [50]. Consequently, we examined and analyzed the variations in shear stress along the surface of the embolus and measured changes in their velocity during movement.

**Fig. 4:**
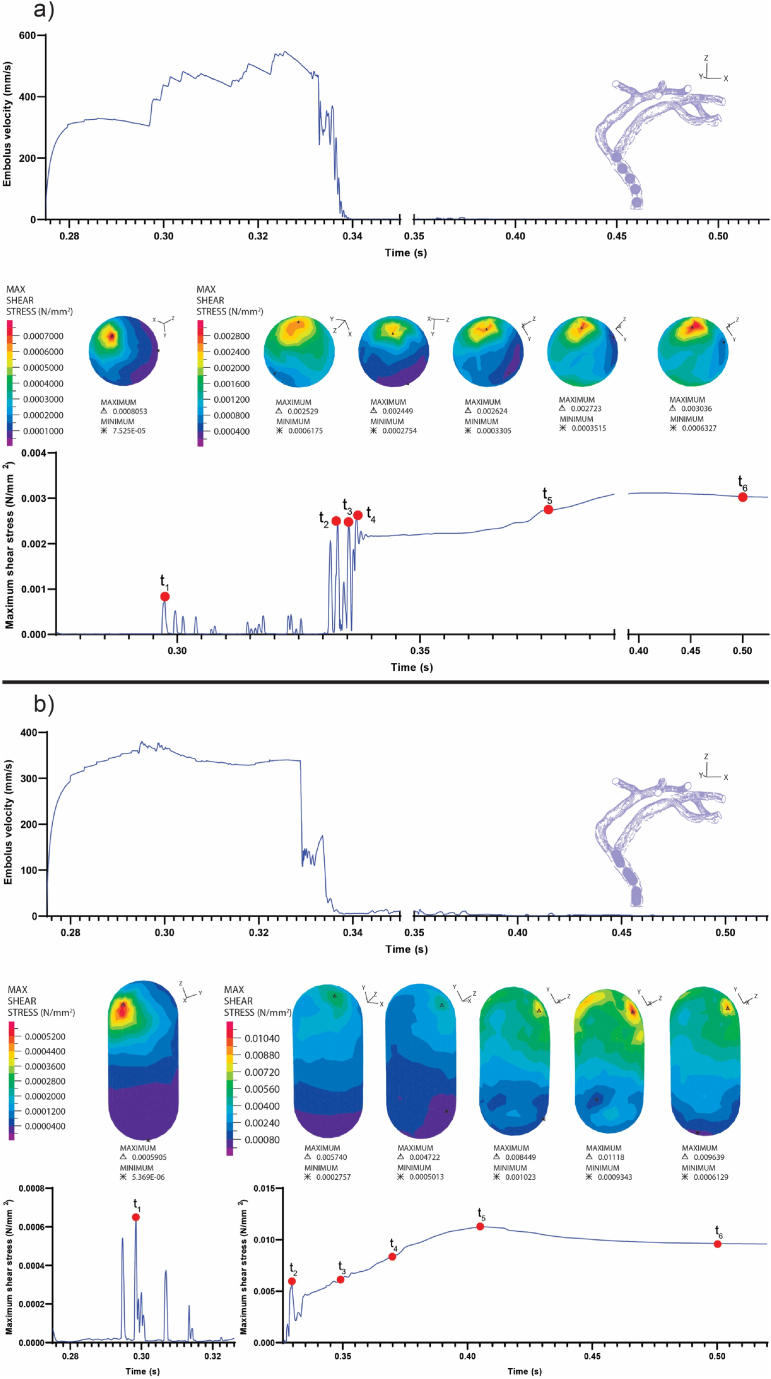
Embolus velocity and maximum shear stress on it: a) S2 Embolus and b) E2 embolus.. (Shear stress contours correspond to specified points in time on the plot. Emboli released at the beginning of systole, and the embolus is depicted within the vasculature to illustrate its position and orientation at different time instances.)

Based on the findings of this study, it is evident that the geometry and size of the embolus are significant factors influencing the embolus's trajectory, movement pattern, and the stress it experiences. Larger emboli exhibit a greater tendency to come to a halt within smaller arteries, as depicted in Fig. 4, thereby causing a hindrance to blood flow and an increase in vascular resistance.

Conversely, smaller emboli, capable of traversing more extensively in smaller branches, manifest more conspicuous effects of geometry. Specifically, the spherical embolus ($S_{1}$) exhibited a tendency to travel in proximity to the artery wall (Fig. S3a), establishing multiple contacts with the wall during its movement, as evidenced by the measured shear stress on its surface (refer to Fig. S3a). In contrast, the elliptical embolus ($E_{1}$) demonstrated a tendency to travel and remain in the center of the artery, resulting in fewer contacts with the artery wall (shown in Fig. S3b). The velocity of the embolus within the artery and the corresponding shear stress, influenced by blood flow, closely aligned with findings from previous studies [20,51]. In all embolus models, releasing them at the peak of systole resulted in higher velocities compared to releasing them at the beginning of systole. Consequently, they encountered elevated shear stress during their collision with the bifurcation of the artery, as depicted in Fig. S4 and Fig. S5 in the supplementary materials. For instance, in the $S_{1}$ model, the shear stress observed during its first collision with the bifurcation differed between scenarios: releasing at the beginning of systole (975.5 Pa) and releasing at the peak of systole (1638.8 Pa).

### 3.2 Visco-hyperelastic properties of embolus

**Fig. 5:**
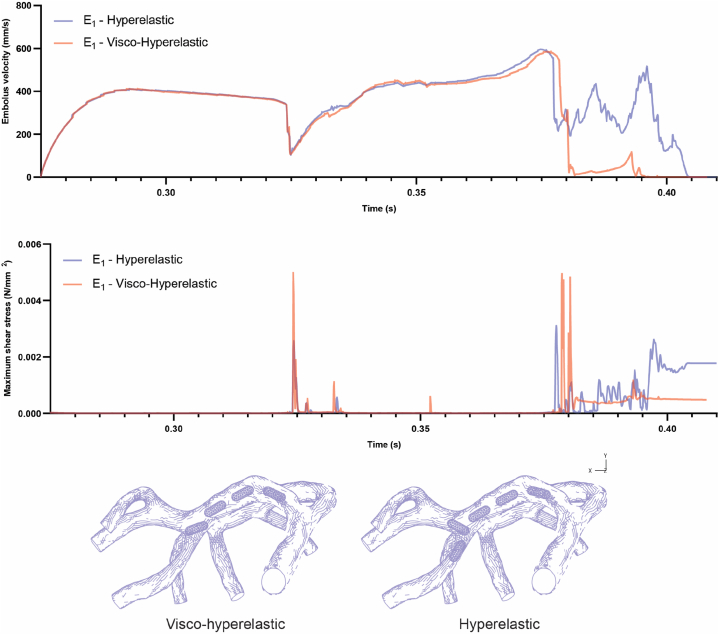
Comparative analysis between visco-hyperelastic and hyperelastic embolus (E_1_) models, focusing on embolus velocity, shear stress magnitude, and embolus trajectory.

The consideration of visco-hyperelastic properties for two geometries ($E_{1}$ and $E_{2}$) led to an exploration of emboli behavior in comparison to hyperelastic models, offering intriguing insights (refer to Fig. 5). When released at the beginning of systole, both models demonstrate similar patterns of speed changes from release to the first collision with the bifurcation. This observation implies that, in this specific scenario, the mechanical properties of the emboli do not exert a significant influence on their speed in the fluid during the initial phase of movement. Nevertheless, a noticeable distinction emerged in the behavior of the visco-hyperelastic model for geometry ($E_{1}$) after it impacted the second bifurcation. Unlike the hyperelastic model, in this scenario, the embolus came to a halt after hitting the second bifurcation and did not proceed into branch number 8 (shown in Fig. 5). This observation indicates that the mechanical properties of the embolus can indeed play a role in influencing its trajectory within the artery.

During the moments of impact, both geometries with visco-hyperelastic properties encountered higher shear stress compared to their counterparts with hyperelastic properties. Specifically, in both the first and second collision moments for the hyperelastic model ($E_{1}$), the highest shear stress on the embolus was 2574 Pa and 3105 Pa, respectively. In contrast, for the visco-hyperelastic model ($E_{1}$), these values rose to 4980.6 Pa and 4944 Pa, respectively. Additionally, the shear stress experienced by the clot during the collision with the bifurcation in the hyperelastic and visco-hyperelastic ($E_{2}$) models was 5740 Pa and 8525.7 Pa, respectively.

This discrepancy in stress is contingent upon the viscosity property of the materials, which exhibit distinct behavior in response to varying strain rates. Faster strain rates tend to make materials behave more like solids (harder), whereas slower strain rates resemble liquid-like behavior [52]. Furthermore, in the visco-hyperelastic model of geometry ($E_{1}$), the embolus avoids entering branch number 8 and consequently experiences less shear stress compared to the embolus that is arrested in the branch (refer to Fig. 5).

**Fig. 6:**
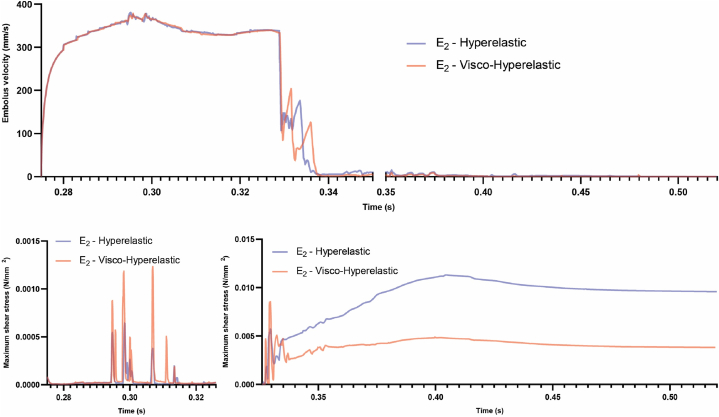
Comparative analysis between visco-hyperelastic and hyperelastic embolus (E_2_) models, focusing on embolus velocity and shear stress magnitude.

In the visco-hyperelastic model of embolus ($E_{2}$), the shear stress on the embolus increases at a slower rate after colliding with the bifurcation wall and coming to a halt, as compared to the hyperelastic model (shown in Fig. 6).

### 3.3 Blood velocity in the branches of MCA

**Fig. 7:**
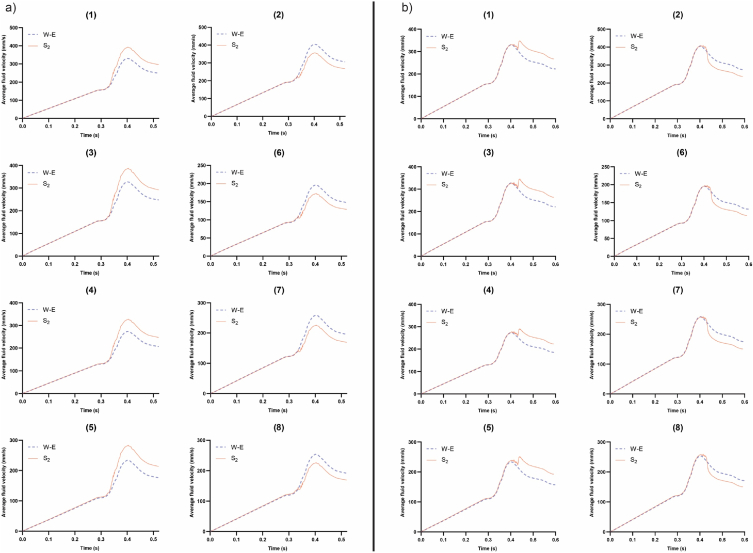
Fluid velocity in the branches of the MCA with the presence of S_2_ embolus: a) embolus released at the beginning of the systole, and b) embolus released at the peak of systole. (Each branch is specified by the number above each plot. W–E: without the presence of emboli).

The examination of blood velocity in each branch of the MCA was conducted with the presence of emboli in the artery. For the $S_{1}$ embolus, its impact on fluid velocity in the MCA branches was negligible, regardless of release times—either at the beginning or at the peak of systole (refer to supplementary material, Fig. S6). In the case of the $E_{1}$ embolus, releasing it at the beginning of systole had little effect on blood velocity in the MCA branches until it reached branch number 8. Stopping at branch 8 resulted in a sudden decrease in fluid velocity in this branch, causing an increase in other branches. However, upon release at the peak of systole, the $E_{1}$ embolus did not impede the flow into branch number 8, resulting in no significant alteration in blood velocity in other branches (supplementary material, Fig. S7). For the $S_{2}$ embolus, after collision with the bifurcation and subsequent halt, the fluid flow path toward branch number 2 and its associated branches narrowed. This led to decreased fluid velocity in these branches and increased fluid velocity in branch number 1 and its daughter branches. Releasing the $S_{2}$ embolus at the peak of systole not only contributed to an overall change in fluid velocity inside the artery but also altered the pattern of fluid velocity changes in the MCA branches (refer to Fig. 7). In the case of the $E_{2}$ embolus, releasing it at the beginning of systole caused an opposite alteration in blood velocity in branches compared to the $S_{2}$ embolus. However, when released at the peak of systole, the changes in blood velocity in branches were similar to the $S_{2}$ embolus, albeit with less profound effects (shown in Fig. S8).

### 3.4 Shear stress on the MCA wall

**Fig. 8:**
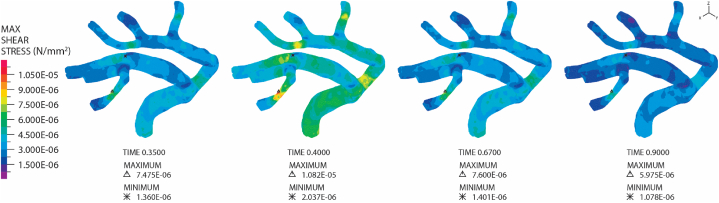
Shear stress on the wall of the MCA at various time points in one pulsation.

Shear stress stands out as one of the most critical hemodynamic mechanical forces influencing the integrity of blood vessel walls. This force arises from the viscous flow of blood over the endothelial cell surface. The arterial and venous vascular systems are exposed to varying degrees of shear stress due to differences in blood flow rates and velocities. Within the normal physiological range, shear stress contributes to the stabilization of blood vessels and facilitates their regeneration following injury. High shear stress is associated with increased survival and quiescence of endothelial cells, platelet alignment with the flow direction, and the secretion of vasodilatory and anticoagulant substances. Conversely, low shear stress or alterations in shear stress direction can lead to endothelial proliferation, apoptosis, changes in vessel shape, secretion of vasoconstrictive substances, and platelet accumulation. Abnormal shear stress distribution plays a pivotal role in conditions like atherosclerosis, hyperplasia, and aneurysm [53]. The shear stress on the artery wall in this study, in the absence of embolism, is depicted in Fig. 8, and its values fall within the physiological range observed in the human body [53]. Subsequently, we will explore the impact of embolism presence on wall shear stress levels.

**Fig. 9:**
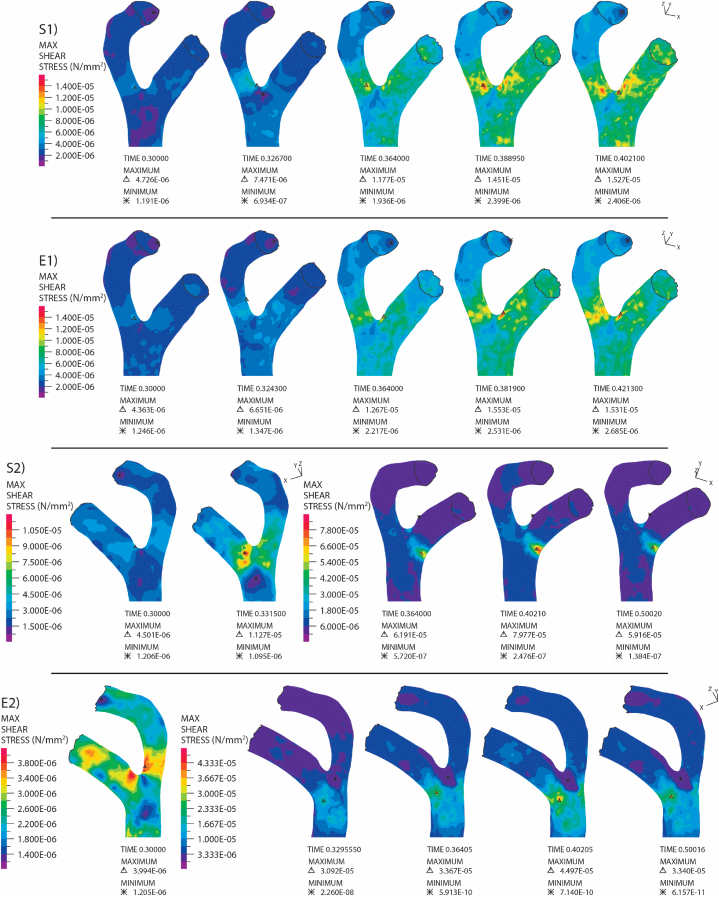
Shear stress on the first part of the MCA with the presence of emboli of different geometries.. (The model of the embolus is specified in each row. All emboli released at the beginning of systole.)

As established in the preceding section, the presence of emboli inside the artery has the potential to either increase or decrease fluid velocity in different branches, thereby significantly influencing the shear stress on the artery wall. To systematically analyze shear stress in our study, our geometry was divided into three sections. The first section encompasses the initial bifurcation, comprising branch number 1 and 2. The second part encompasses branches with numbers 3, 4, and 5, while the last part includes branches numbered 6, 7, and 8. Based on the analysis of Fig. 9 and Figs. S9–S10, the effects of the presence of $S_{1}$ and $E_{1}$ emboli were not significantly different. When the $E_{1}$ embolus was released at the beginning of systole, it halted at branch number 8, decreasing the flow within that branch, and subsequently increasing fluid velocity in other branches. This led to a noticeable contrast with the situation involving the $S_{1}$ embolus, as the increased fluid velocity in other branches resulted in an elevation of the magnitude of shear stress they experienced. This change is distinguishable when compared to the scenario where the $S_{1}$ embolus was released inside the artery. Evaluation of branch number 8 in the third part of the artery revealed a substantial increase in the magnitude of shear stress attributed to the presence of emboli ($S_{1}$ and $E_{1}$) in that area. Specifically, for the case of $E_{1}$ embolus presence, the magnitude of shear stress was 27.59 Pa, while for the $S_{1}$ embolus presence, it was 16.5 Pa. A significant decrease in the magnitude of shear stress in branch number 8 was evident in the case of $E_{1}$ embolus presence, attributed to its halt in that branch and obstruction of fluid flow inside it.

When both emboli were released at the peak of systole, the changes in the magnitude of shear stress on the artery wall were not as significant as when released at the beginning of systole (refer to Figs. S11–S13 in supplementary materials). In the case of $E_{1}$ embolus released at the peak of systole, after colliding with the first bifurcation, it stopped in that area, leading to a shift in the pattern of shear stress and a change in the area experiencing the maximum magnitude of shear stress. Another notable observation was the decrease in the amount of shear stress in that area, measured at 4.893e-5 Pa (refer to Fig. S11 in supplementary materials).

Analyzing the velocity plots revealed that when the $S_{2}$ embolus was released at the beginning of systole, it significantly influenced fluid velocity within the branches. There was a notable decrease in fluid velocity in branch number 2 and its associated daughter branches, leading to a decrease in the magnitude of shear stress in those areas. Conversely, the opposite effect occurred in branch number 1 and its daughter branches (shown in Fig. 9). The effects of the $E_{2}$ embolus was less significant compared to the $S_{2}$ embolus. In both cases, when they halted after colliding with the first bifurcation, a noticeable change occurred in the minimum and maximum stress in that area. Specifically, the minimum and maximum magnitudes of shear stress in the area of the first bifurcation were 0.1384 Pa and 79.77 Pa for the case of $S_{2}$ embolus presence, and 6.157e-5 Pa and 44.97 Pa for the case of $E_{2}$ embolus presence (refer to Fig. 9). In the scenario where these emboli were released at the peak of systole, both obstructed and narrowed fluid flow toward branch number 2 and its associated branches. Consequently, the magnitude of shear stress experienced by these branches decreased, while the shear stress magnitude in the other branches increased. The position of the artery experiencing the minimum and maximum magnitudes of shear stress changed significantly, as depicted in Figs. S11–S13 in the supplementary material.

### 3.5 Non-Newtonian behavior of the blood

To study the effects of non-Newtonian behavior of blood, simulations were conducted to simulate the trajectory and movement of $E_{1}$ and $E_{2}$ emboli within the MCA. In each study, the embolus was released at the beginning of the systole.

#### 3.5.1 Shear stress on the emboli and their velocity

**Fig. 10:**
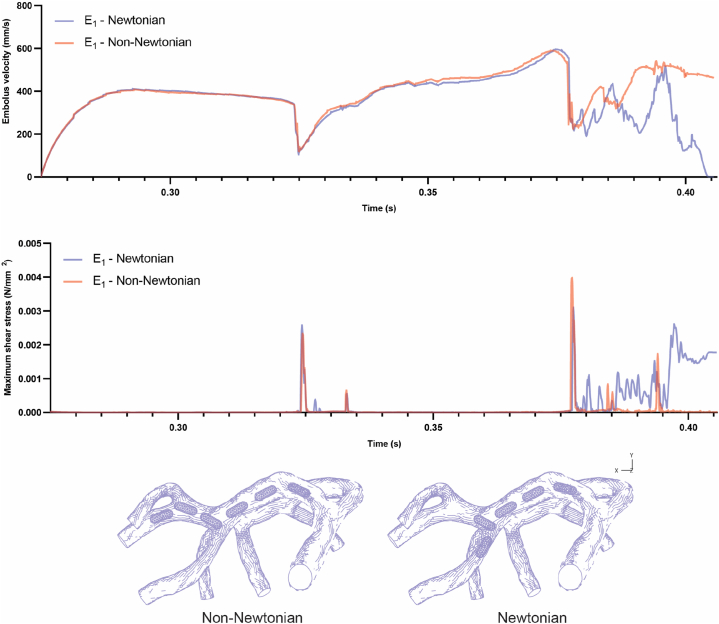
Comparative analysis between non-Newtonian and Newtonian blood with the presence of E_1_ embolus, focusing on embolus velocity, shear stress magnitude, and embolus trajectory.

As illustrated in Fig. 10, the velocity of the $E_{1}$ embolus traveling inside the blood exhibited similar patterns with both Newtonian and non-Newtonian behavior until it collided with the first bifurcation. Subsequently, a slight difference in velocity between the two models was observed until reaching the second bifurcation. Notably, a distinct behavior emerged after the collision with the second bifurcation when the $E_{1}$ embolus was within blood with non-Newtonian features. In this case, it did not enter branch number 8 but continued its trajectory towards branch number 6 and 7, eventually entering branch number 7.

Analysis of the shear stress plot in Fig. 10 revealed that the magnitude of shear stress on the $E_{1}$ embolus in both models was almost similar until the collision with the first bifurcation. However, during the collision with the second bifurcation, the magnitude of shear stress experienced by the $E_{1}$ embolus within blood with non-Newtonian behavior was higher compared to the Newtonian model. In contrast to the $E_{1}$ embolus within the Newtonian blood, which became lodged inside branch number 8 and experienced constant shear stress, the $E_{1}$ embolus within the non-Newtonian blood had an additional collision with the bifurcation between branch number 6 and 7. By continuing its movement inside branch number 7, the magnitude of shear stress on it decreased.

**Fig. 11:**
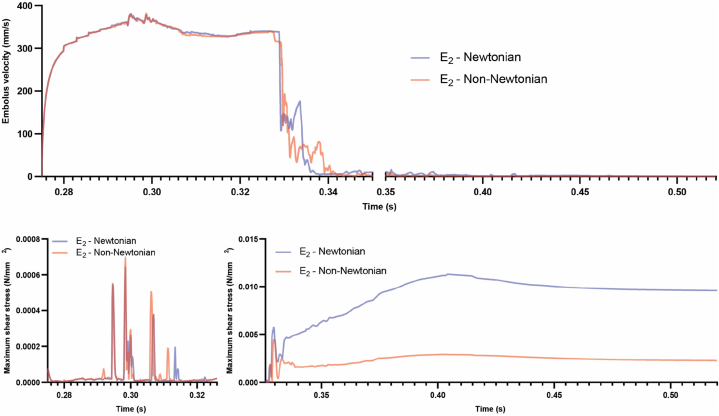
Comparative analysis between non-Newtonian and Newtonian blood with the presence of E_2_ embolus, focusing on embolus velocity and shear stress magnitude.

In the case of the $E_{2}$ embolus, the velocity of the embolus was nearly identical in both Newtonian and non-Newtonian blood. As depicted in Fig. 11, the magnitude of shear stress in both models remained almost similar until the collision with the first bifurcation. After the collision and subsequent halt in that area, the magnitude of shear stress on the embolus fluctuated, increasing, and decreasing in response to changes in fluid velocity. Notably, in the non-Newtonian blood, the magnitude of shear stress experienced by the embolus was significantly lower than in the case with Newtonian blood.

#### 3.5.2 Fluid velocity and pressure gradient in the presence of an embolus

**Fig. 12:**
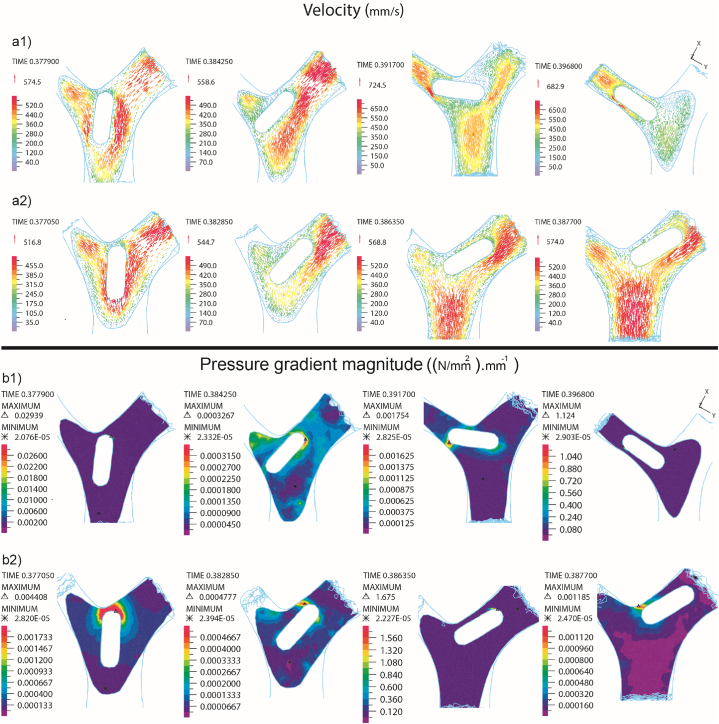
Comparative analysis between non-Newtonian (a2, b2) and Newtonian (a1, b1) blood with the presence of E_1_ embolus, focusing on the velocity vectors around the embolus (a1, a2) and the pressure gradient around the embolus (b1, b2).

The comparison of fluid velocity vectors between Newtonian and non-Newtonian fluid states in the second bifurcation of the artery with the presence of embolism ($E_{1}$) is presented in Fig. 12a1-a2. As we move beyond the main branches of the artery and the diameter of the daughter arteries decreases, both fluid velocity and strain rate decrease. The presence of a clot in this area also influences the extent of this decrease. Notably, a reduction in strain rate results in an increase in the viscosity of the non-Newtonian fluid, subsequently leading to a decrease in fluid velocity [40]. This change in velocity has an impact on the pressure gradient around the embolus and may lead to alterations in the direction of its movement. The impact of Newtonian and non-Newtonian fluids on the pressure gradient around the clot in the bifurcation is illustrated in Fig. 12b1-b2. As previously discussed, fluid velocity was higher in the Newtonian state compared to the non-Newtonian state, resulting in differing pressure distributions around the embolus in these two models. In both models, up until the embolus reached the bifurcation, the pressure in front of the embolus was elevated, decelerating its speed. In the Newtonian and non-Newtonian fluid states, this pressure was measured at 29.39 MPa and 4.408 MPa, respectively.

Following the impact, the tip of the embolus in both models tilted towards branches number 6 and 7. In the Newtonian fluid model, there was a higher pressure applied to the side area of the embolus, causing it to incline towards branch number 8, eventually leading the embolus to enter branch number 8. Conversely, in the non-Newtonian fluid, the pressure applied to the side area of the embolus was lower, allowing the clot to continue moving with the flow and towards branches 6 and 7.

**Fig. 13:**
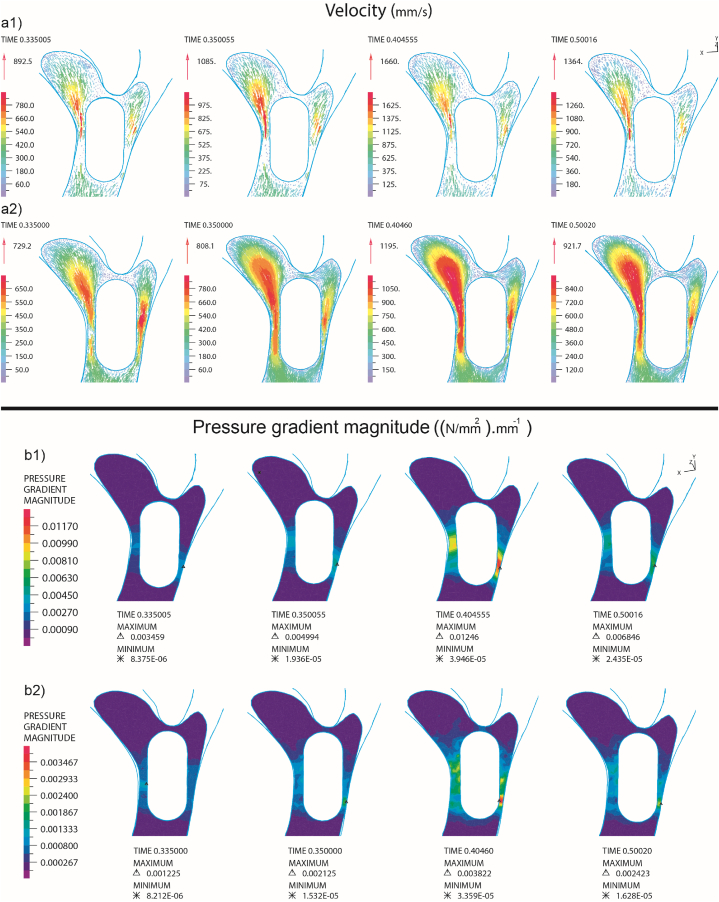
Comparative analysis between non-Newtonian (a2, b2) and Newtonian (a1, b1) blood with the presence of E_2_ embolus, focusing on the velocity vectors around the embolus (a1, a2) and the pressure gradient around the embolus (b1, b2).

As mentioned in the previous section, the magnitude of shear stress experienced by the $E_{2}$ embolus after collision with the bifurcation was lower when it was within non-Newtonian blood compared to Newtonian blood. By analyzing the blood velocity and pressure gradient around the embolus in both cases, it is evident that in non-Newtonian blood, the presence of the clot has reduced the shear rate, leading to an increase in fluid viscosity. This increase in viscosity has resulted in a decrease in fluid velocity around the embolus compared to Newtonian fluid, as shown in Fig. 13a1-a2.

With the decrease in fluid velocity, the pressure exerted by the fluid on the embolus has also decreased, and this pressure differential is illustrated in Fig. 13b1-b2 for both Newtonian and non-Newtonian fluid models. Consequently, in the non-Newtonian fluid model, the embolus experienced less pressure and was pressed against the wall with reduced force, leading to lower stress on the embolus. Specifically, the pressure values in the Newtonian and non-Newtonian fluid models were 29.39 MPa and 4.408 MPa, respectively.

### 3.6 Embolus rupture

Investigating the likelihood of emboli breaking or fragmenting is crucial, especially considering the higher risk posed by emboli obstructing larger arteries. In a study by Ref. [54], the structural properties of emboli from stroke patients and the force required to dislodge them were explored, reporting yield stress and fracture values ranging from 63 kPa to 2396 kPa.

In our study, the maximum von Mises stress values for the emboli (Table 6) are lower than the reported values, suggesting a very low probability of emboli breaking during movement, impact, and after coming to a stop. Another study by Ref. [55] found that emboli with cracks are more prone to rupture, establishing a criterion for embolus cracking at a shear stress level of 1 kPa. Our analysis of shear stress values indicates the potential for embolus cracking upon collision with the artery wall and when lodged within the artery. Larger emboli, halted and in contact with the artery wall, experience prolonged exposure to shear stresses exceeding 1 kPa compared to smaller emboli, increasing the likelihood of cracking and disintegration.

**Table 6:** Maximum von Mises stress values for all models in our study.

Model	Geometry	Embolus property	Blood property	Release time (systole)	Maximum Von Mises stress (Kpa)
1	S_1_	Hyperelastic	Newtonian	Beginning	4.13
2	S_1_	Hyperelastic	Newtonian	Peak	2.98
3	E_1_	Hyperelastic	Newtonian	Beginning	5.63
4	E_1_	Hyperelastic	Newtonian	Peak	10.8
5	S_2_	Hyperelastic	Newtonian	Beginning	5.55
6	S_2_	Hyperelastic	Newtonian	Peak	9.94
7	E_2_	Hyperelastic	Newtonian	Beginning	21.83
8	E_2_	Hyperelastic	Newtonian	Peak	14.59
9	E_1_	Visco-hyperelastic	Newtonian	Beginning	9.41
10	E_2_	Visco-hyperelastic	Newtonian	Beginning	14.68
11	E_1_	Hyperelastic	non-Newtonian	Beginning	7.06
12	E_2_	Hyperelastic	non-Newtonian	Beginning	7.77

## 4 Limitation and future works

In this study, we simplified the artery wall as a rigid structure to streamline computational costs. However, future investigations could benefit from considering the mechanical properties of the artery wall, enabling a more comprehensive exploration of its interactions with blood and associated deformations. Additionally, the scope of research could be expanded to include different types of emboli, such as gas emboli, providing a more nuanced understanding of their impact. Furthermore, future studies might delve into the simultaneous presence of multiple emboli within the artery, unraveling the complexities of their interactions and potential complications. An intriguing avenue for exploration involves studying the effects of thrombolysis drugs, commonly used to lyse emboli. Analyzing their impact on shear stress applied to both emboli and the artery, along with other relevant parameters, could offer valuable insights for medical practitioners. It's noteworthy that certain drugs may influence fluid viscosity, adding an extra layer of complexity to the investigation.

## 5 Conclusion

The primary objective of this study was to conduct a comprehensive evaluation of embolism phenomena and simulate the movement of emboli with varying properties within the MCA. Leveraging MRA images and computational fluid dynamics, we developed a robust model to examine the thromboembolism phenomenon in the MCA. Our findings underscored the significant impact of the embolus geometry on its trajectory and movement within the artery. Additionally, the release time of the embolus emerged as a crucial parameter influencing both the magnitude of stress experienced by the embolus and its trajectory within the artery. Introducing the concept of the embolus as a visco-hyperelastic material revealed noteworthy distinctions compared to the hyperelastic model. Furthermore, our study highlighted the substantial effects of considering the non-Newtonian behavior of blood on the stresses exerted on the emboli and their trajectory during collisions. However, for the overall simulation, the non-Newtonian model may not significantly affect the results unless collisions occur. In addition to these insights, our research enhanced understanding of fluid hemodynamics within the MCA, particularly in scenarios where a segment of the cerebral artery is obstructed due to the presence of an embolus.

## CRediT authorship contribution statement

**Pouria Talebibarmi:** Conceptualization, Formal analysis, Investigation, Methodology, Resources, Software, Validation, Writing – original draft. **Bahman Vahidi:** Conceptualization, Data curation, Funding acquisition, Project administration, Resources, Supervision, Validation, Writing – review & editing. **Mahtab Ebad:** Visualization, Writing – original draft.

## Declaration of competing interest

The authors have no conflicts of interest related to this work.
