# Supplementary material for: In silico analysis of embolism in cerebral arteries using fluid-structure interaction method
Source: Heliyon. 2024 Apr 27;10(9):e30443. doi: 10.1016/j.heliyon.2024.e30443 (PMC11077041; doi:10.1016/j.heliyon.2024.e30443)
Supplement: Multimedia component 1 [file mmc1.docx]

Heliyon

Electronic Supplementary Information

In silico analysis of embolism in cerebral arteries using fluid-structure interaction method

Pouria Talebibarmi, Bahman Vahidi^*^, Mahtab Ebad

*Division of Biomedical Engineering, Department of Life Science Engineering, Faculty of New Sciences and Technologies, University of Tehran, Tehran, Iran*

*Corresponding author:

Bahman Vahidi ([bahman.vahidi@ut.ac.ir](mailto:bahman.vahidi@ut.ac.ir))


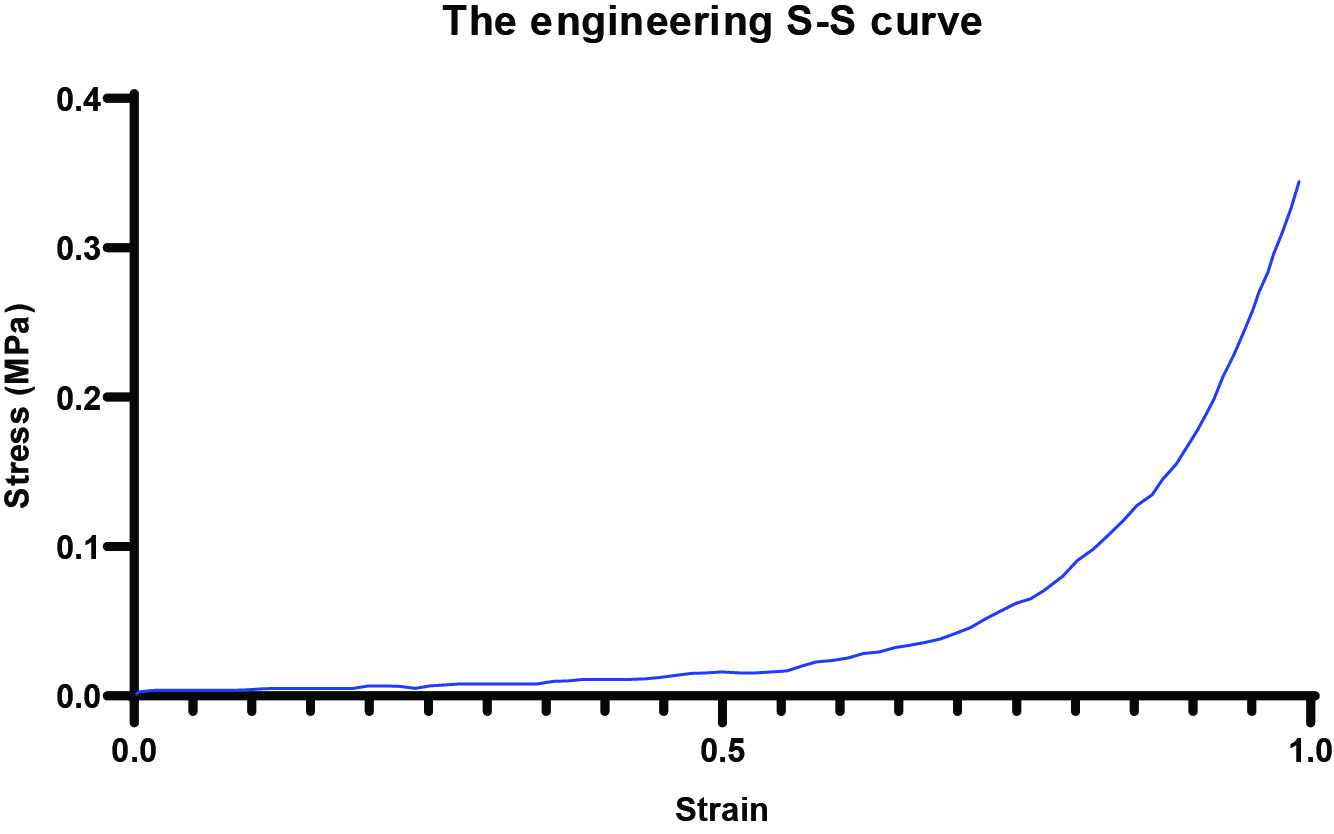


Fig. S 1. Stress-strain curve employed to define the Mooney-Rivlin model [1].


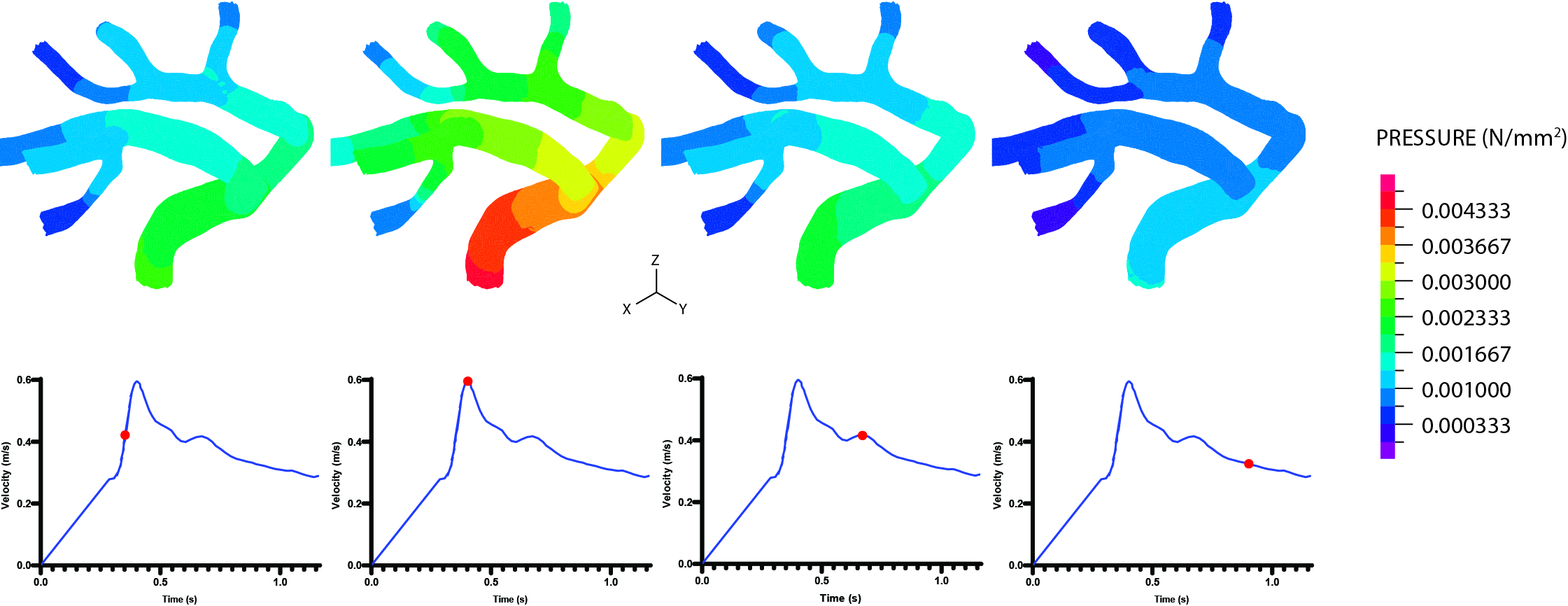


Fig. S 2. Pressure distribution in MCA at various time points in one pulsation [2].


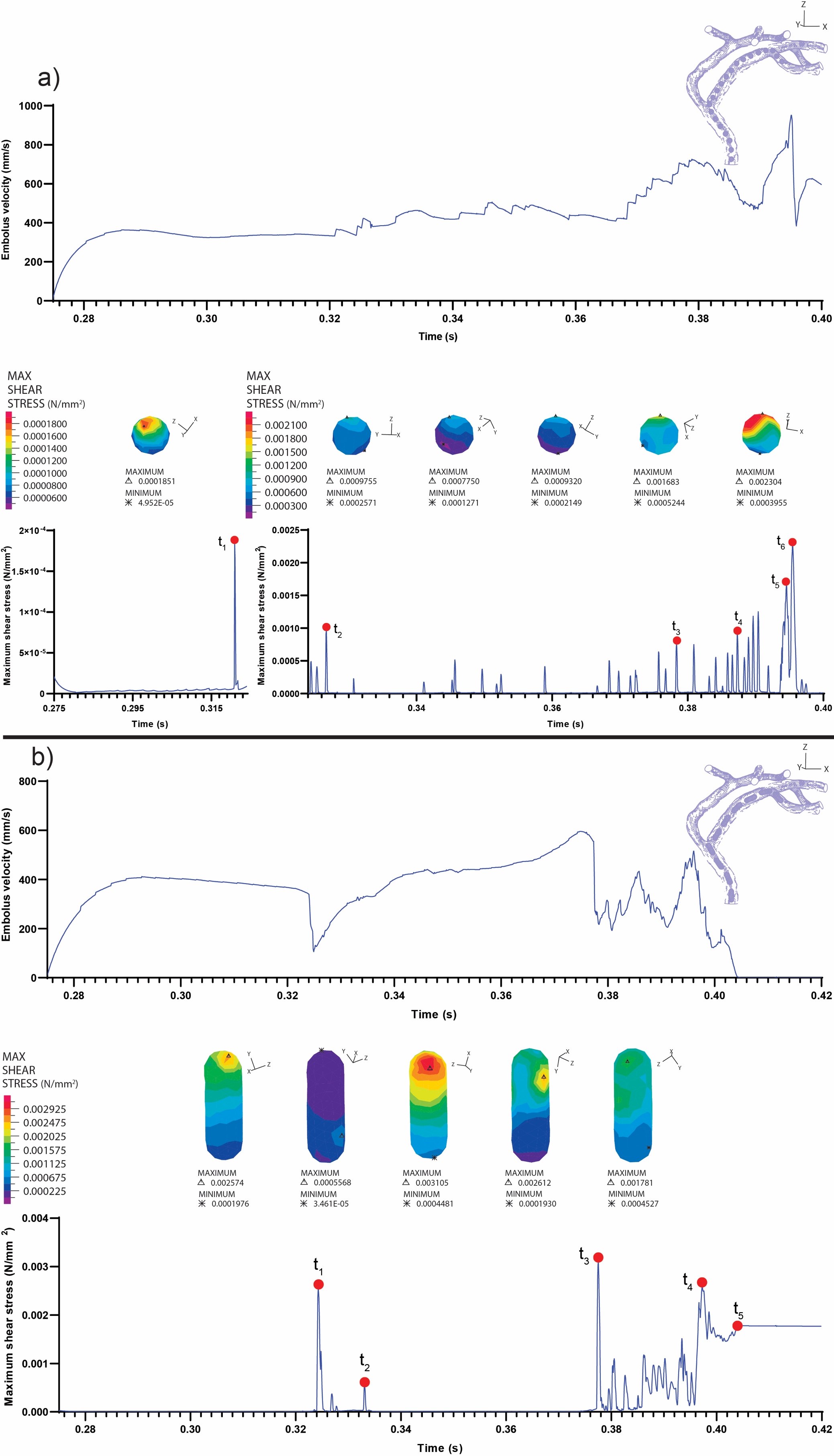


Fig. S 3. Embolus velocity and maximum shear stress on it: a) S1 Embolus and b) E1 embolus. (Shear stress contours correspond to specified points in time on the plot. Emboli released at the beginning of the systole, and the embolus is depicted within the vasculature to illustrate its position and orientation at different time instances.)


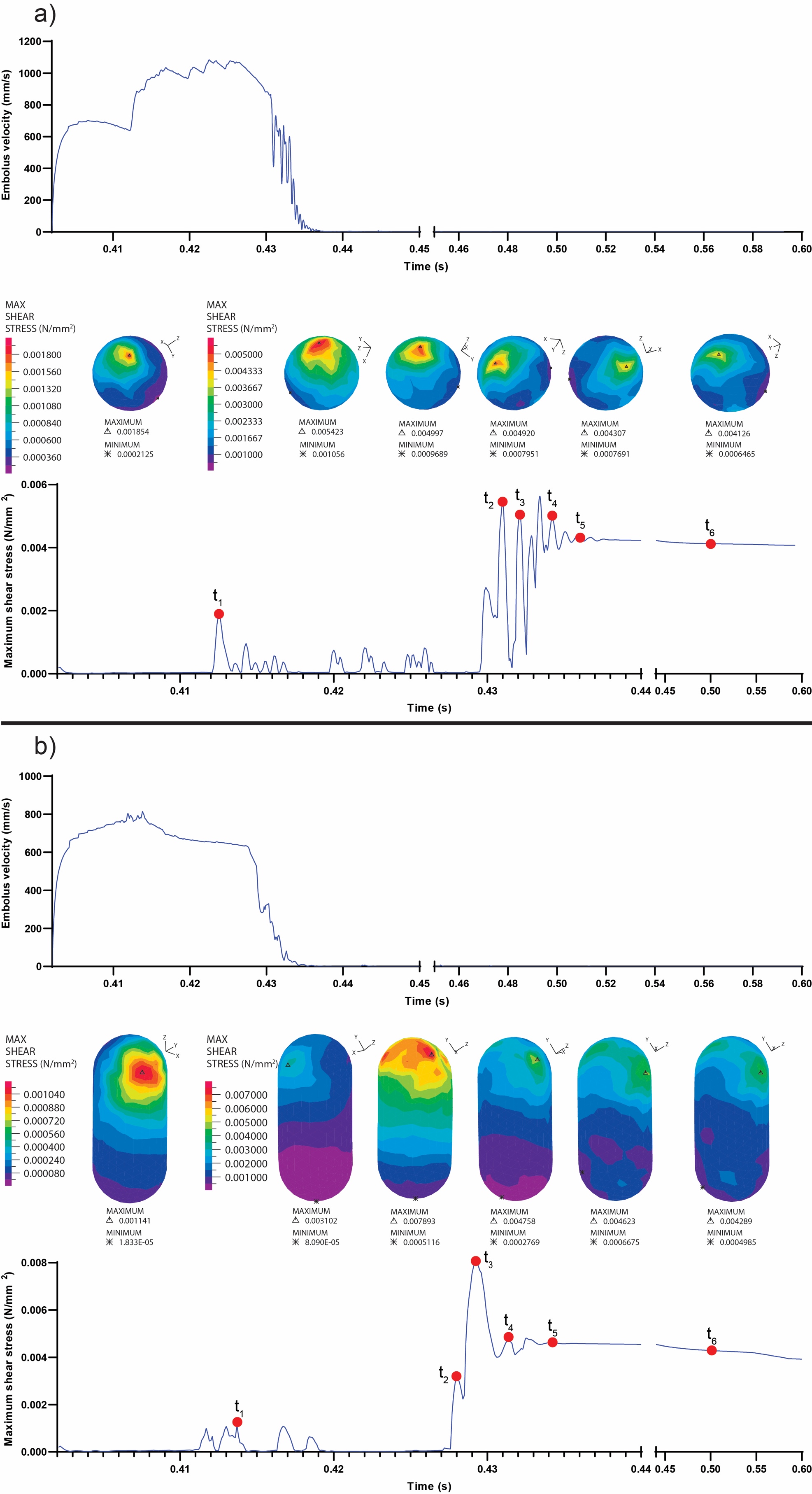


Fig. S 4. Embolus velocity and maximum shear stress on it: a) S2 Embolus and b) E2 embolus. (Shear stress contours correspond to specified points in time on the plot Emboli released at the peak of the systole, and the embolus is depicted within the vasculature to illustrate its position and orientation at different time instances.)


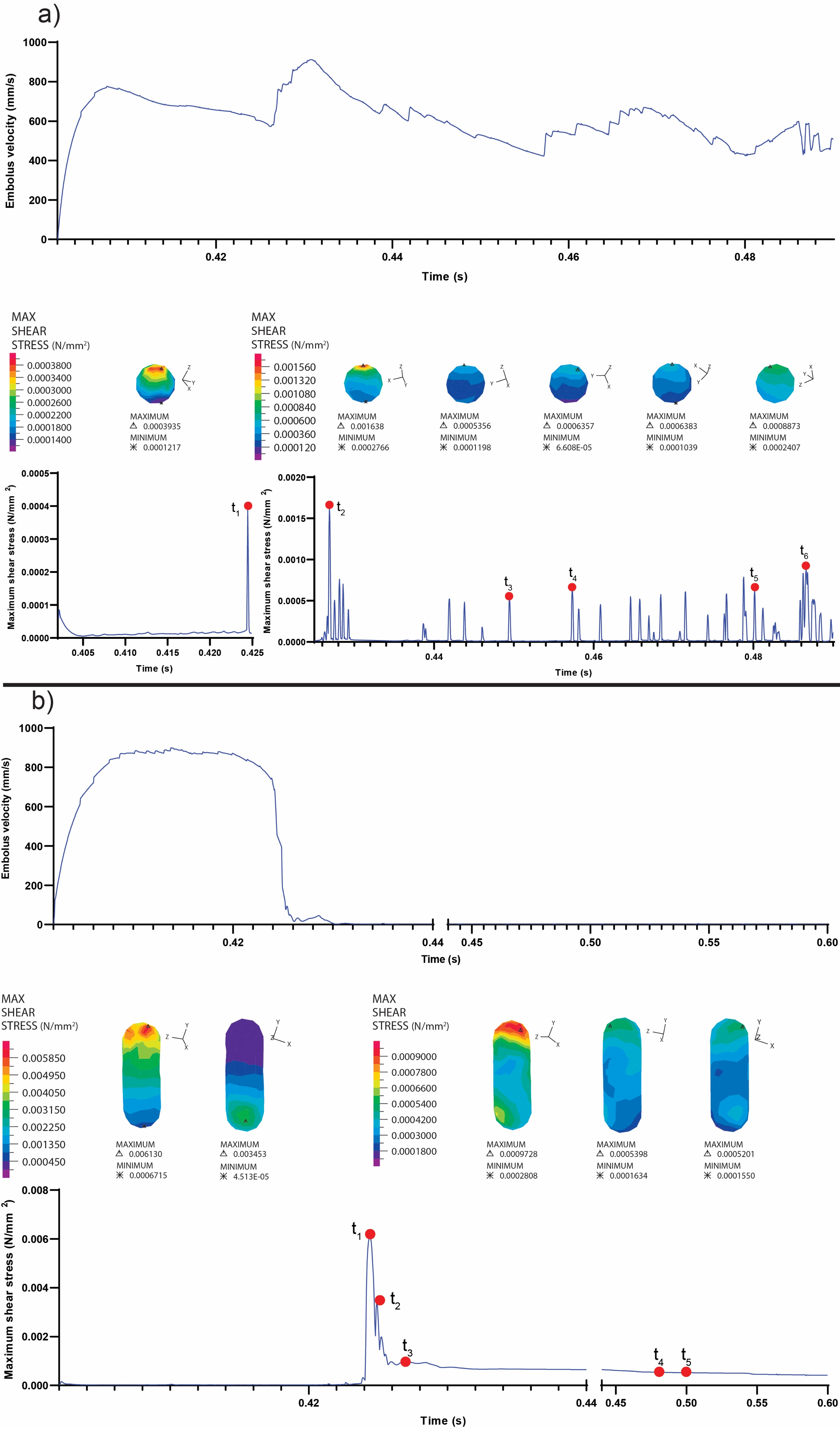


Fig. S 5. Embolus velocity and maximum shear stress on it: a) S1 Embolus and b) E1 embolus. (Shear stress contours correspond to specified points in time on the plot. Emboli released at the peak of the systole, and the embolus is depicted within the vasculature to illustrate its position and orientation at different time instances.)


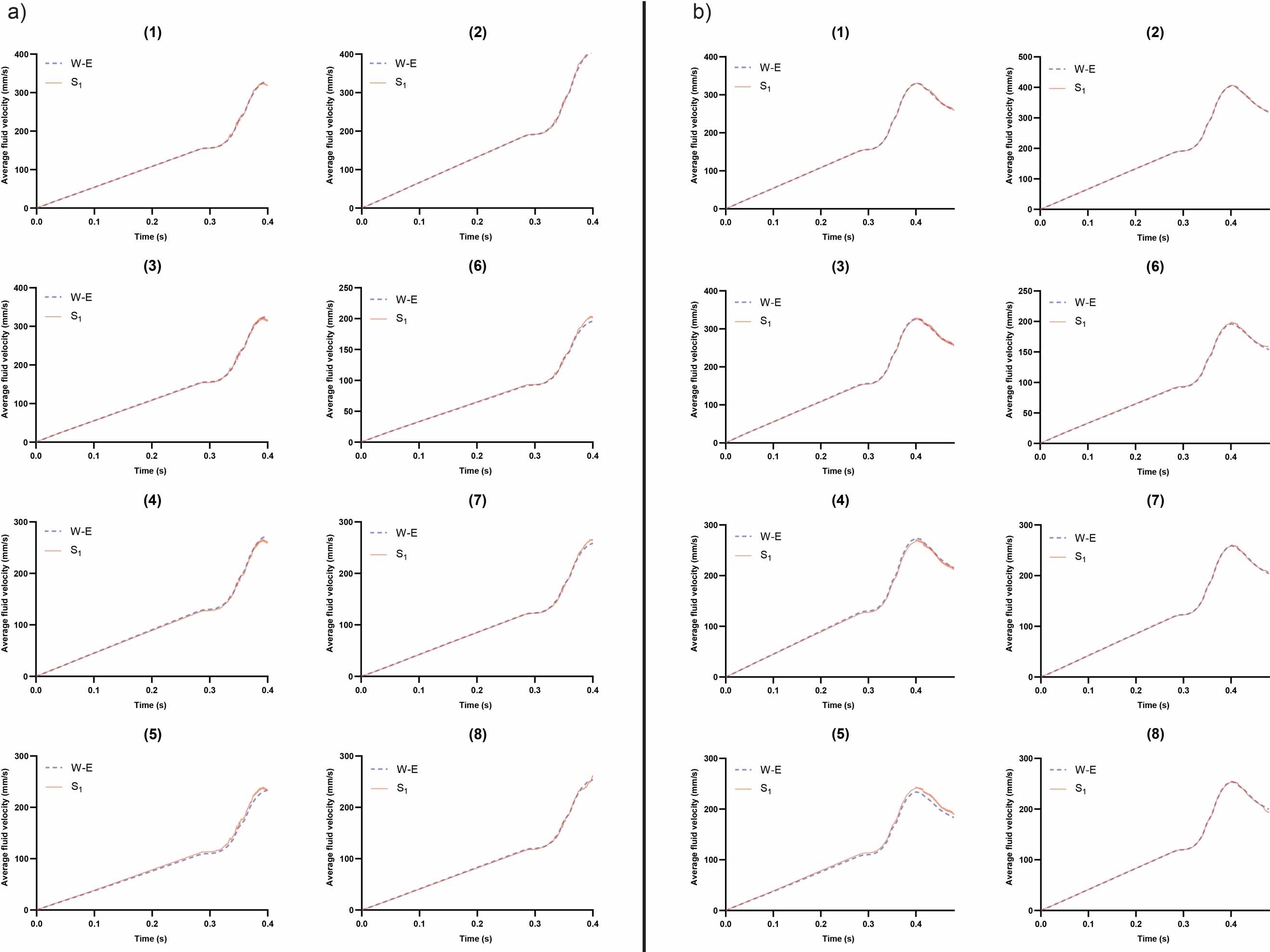


Fig. S 6. Fluid velocity in the branches of the MCA with the presence of S_1_ embolus: a) embolus released at the beginning of the systole, and b) embolus released at the peak of systole. (Each branch is specified by the number above each plot. W-E: without the presence of emboli)


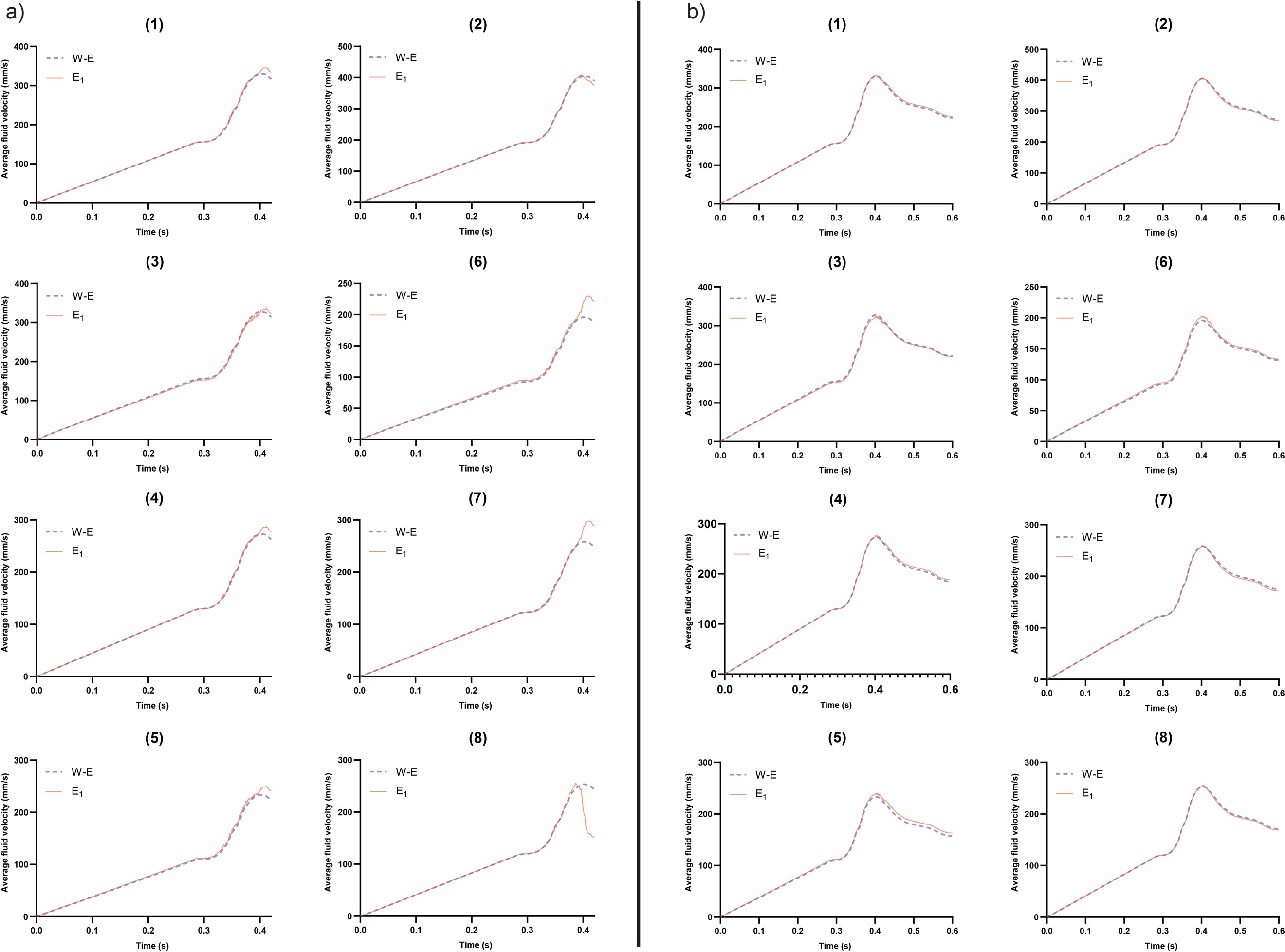


Fig. S 7. Fluid velocity in the branches of the MCA with the presence of E_1_ embolus: a) embolus released at the beginning of the systole, and b) embolus released at the peak of systole. (Each branch is specified by the number above each plot. W-E: without the presence of emboli)


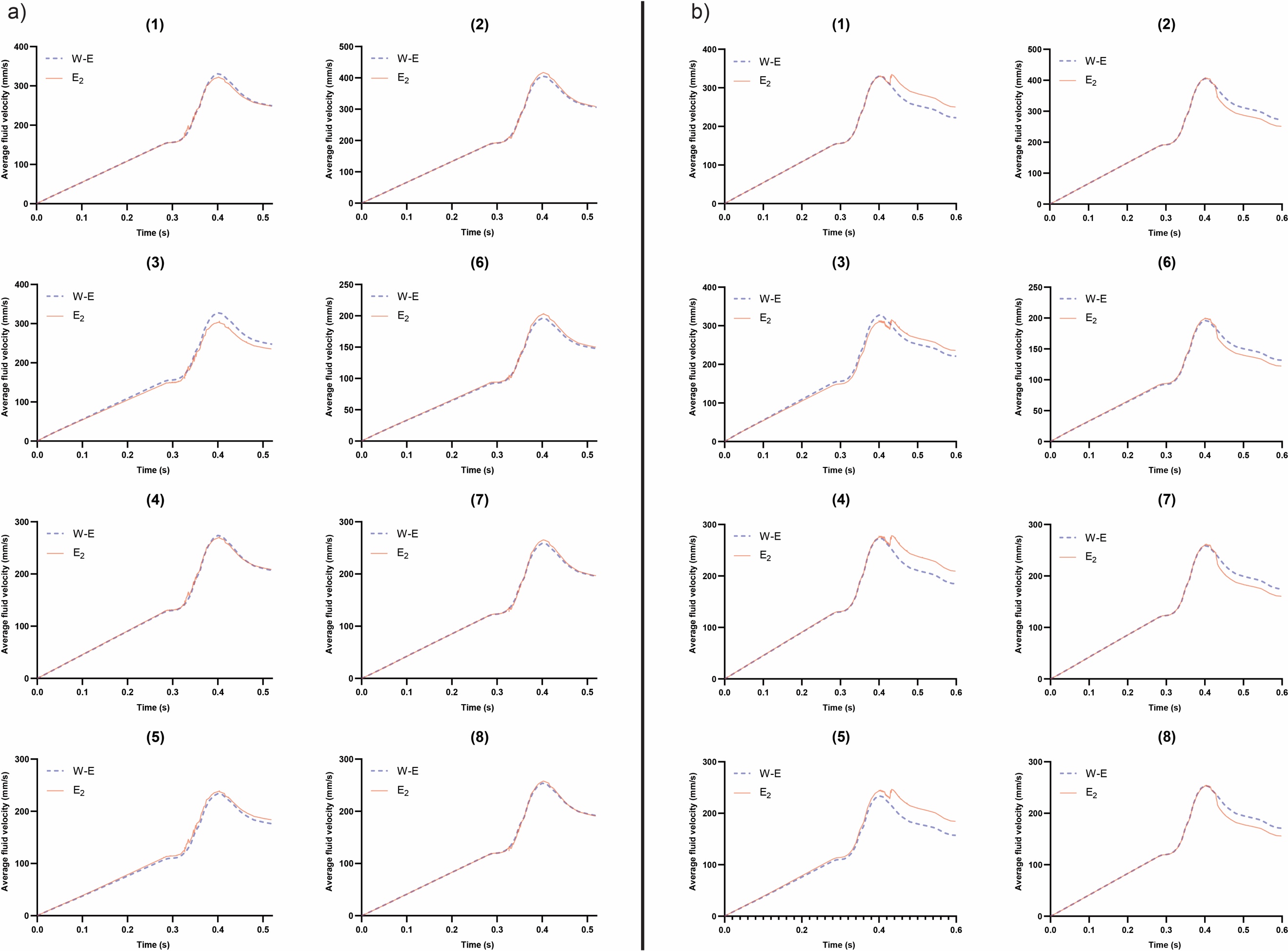


Fig. S 8. Fluid velocity in the branches of the MCA with the presence of E_2_ embolus: a) embolus released at the beginning of the systole, and b) embolus released at the peak of systole. (Each branch is specified by the number above each plot. W-E: without the presence of emboli)


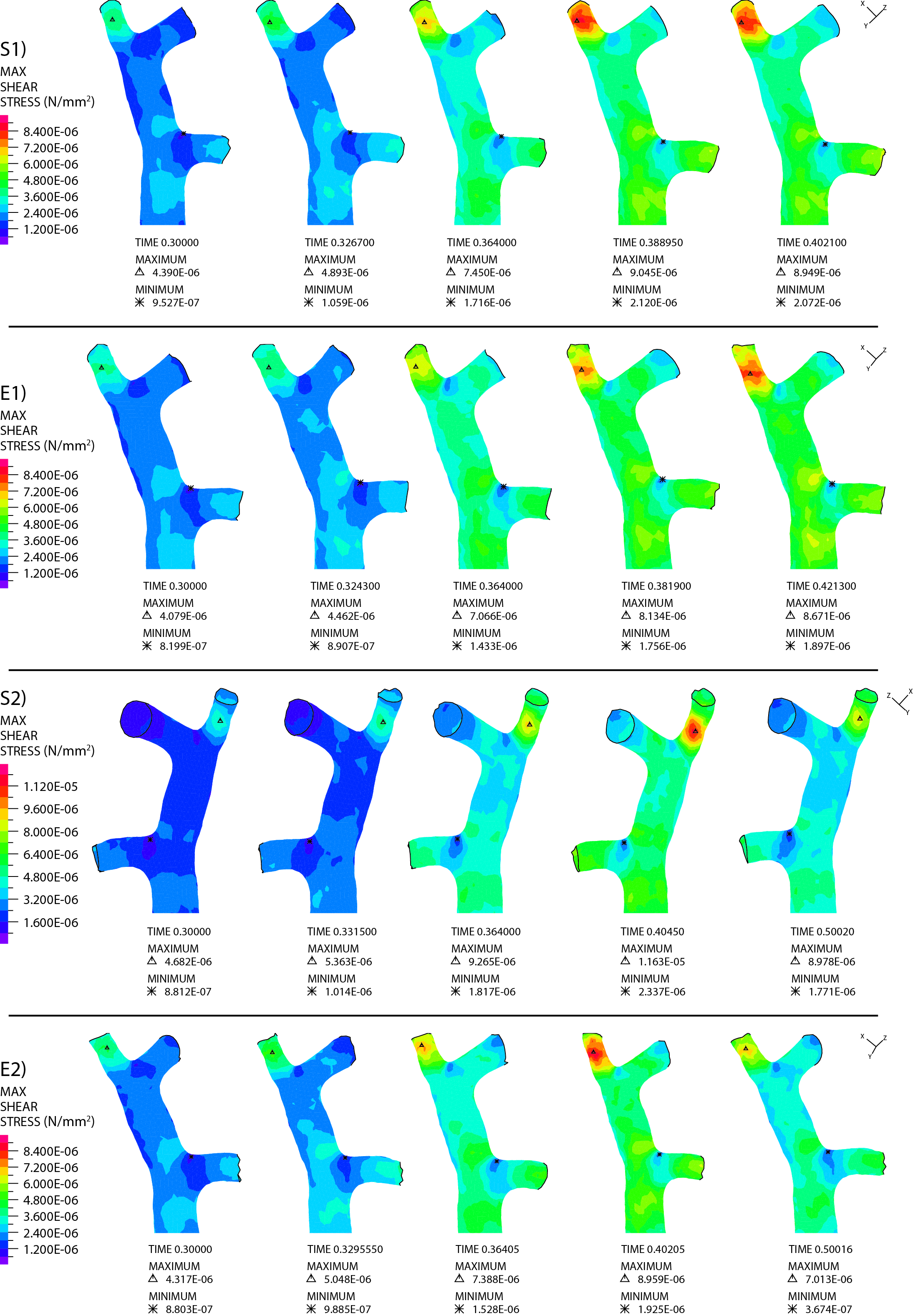


Fig. S 9. Shear stress on the second part of the MCA with the presence of emboli of different geometries. (The model of the embolus is specified in each row. All emboli released at the beginning of systole.)


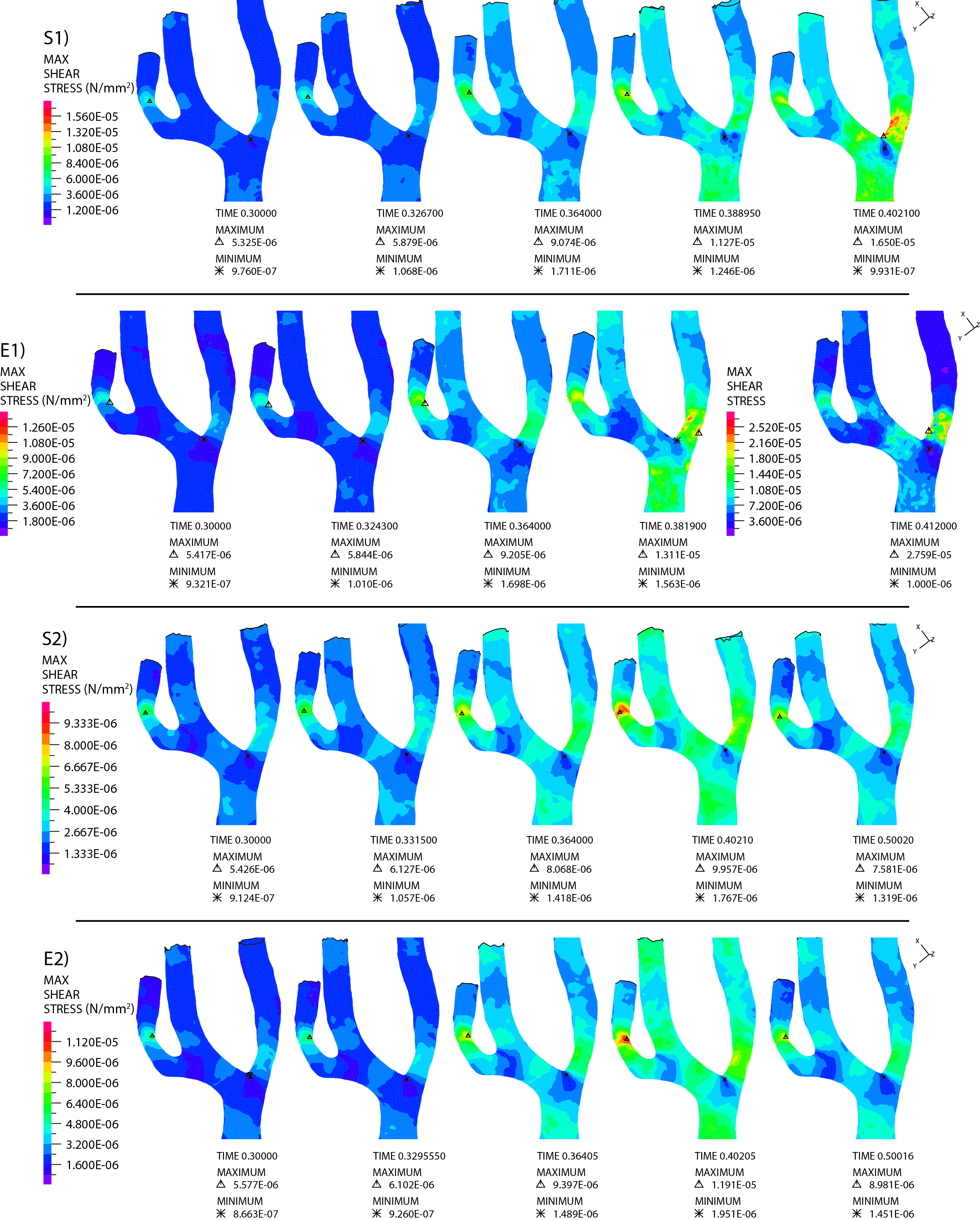


Fig. S 10. Shear stress on the third part of the MCA with the presence of emboli of different geometries. (The model of the embolus is specified in each row. All emboli released at the beginning of systole.)


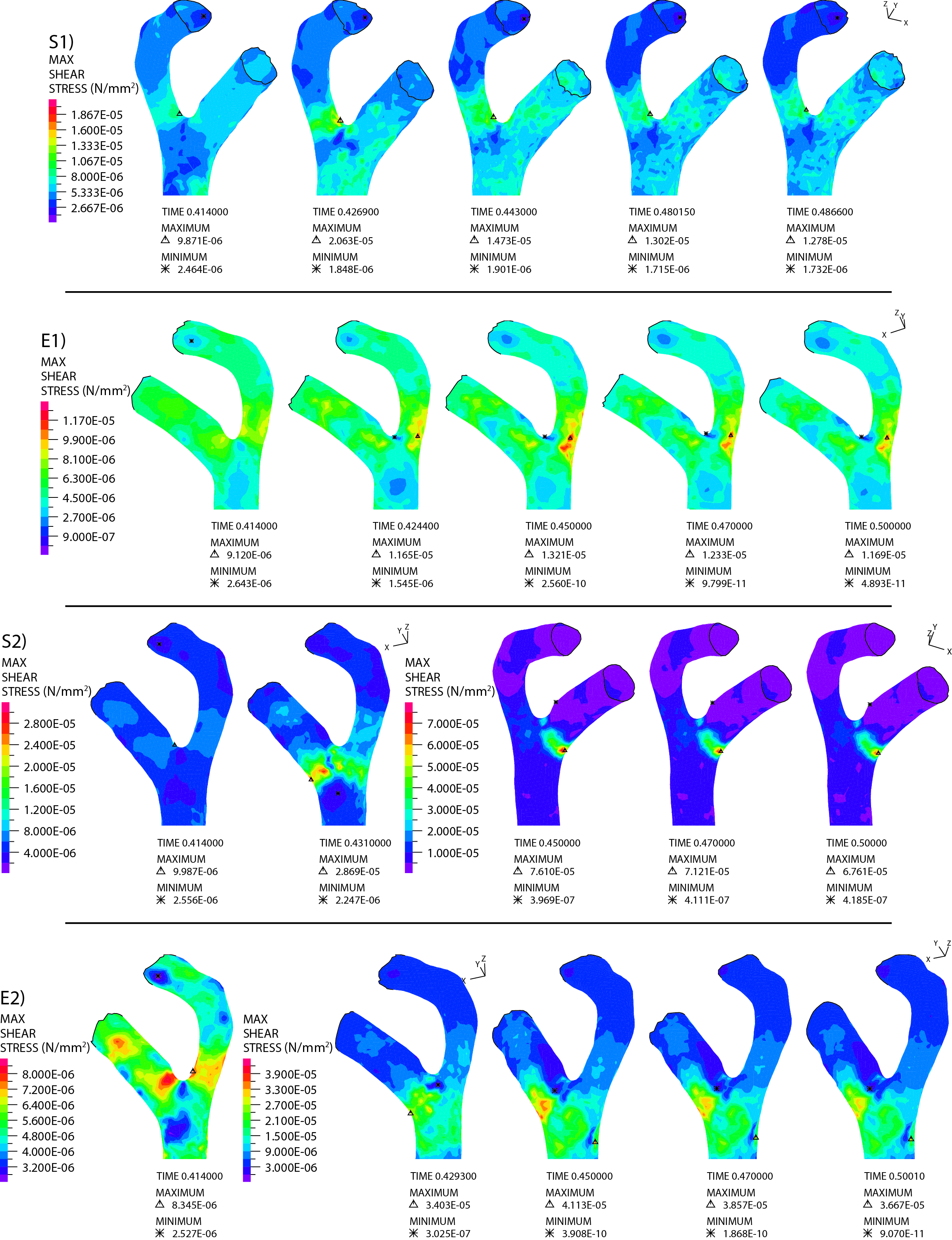


Fig. S 11. Shear stress on the first part of the MCA with the presence of emboli of different geometries. (The model of the embolus is specified in each row. All emboli released at the peak of systole.)


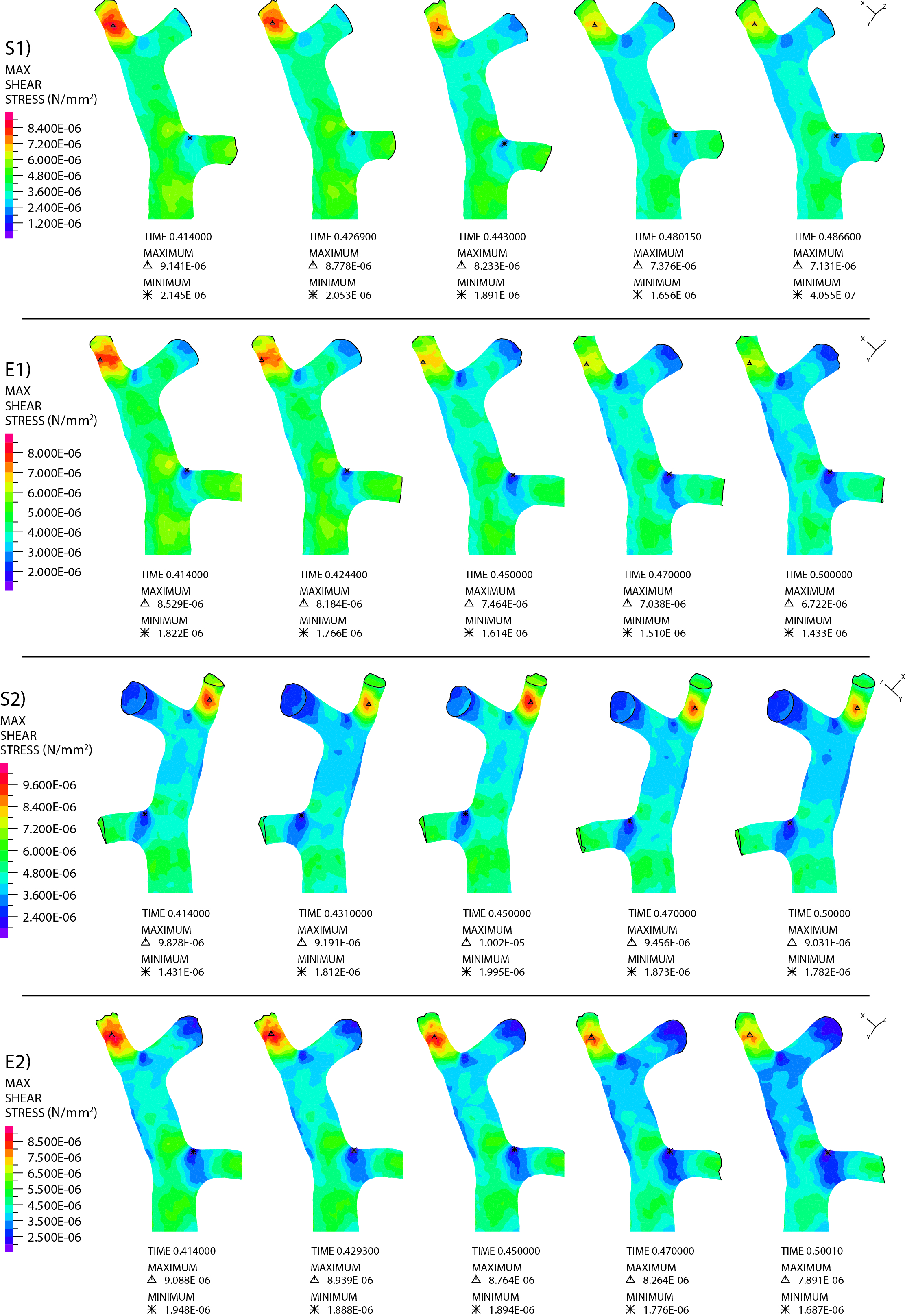


Fig. S 12. Shear stress on the second part of the MCA with the presence of emboli of different geometries. (The model of the embolus is specified in each row. All emboli released at the peak of systole.)


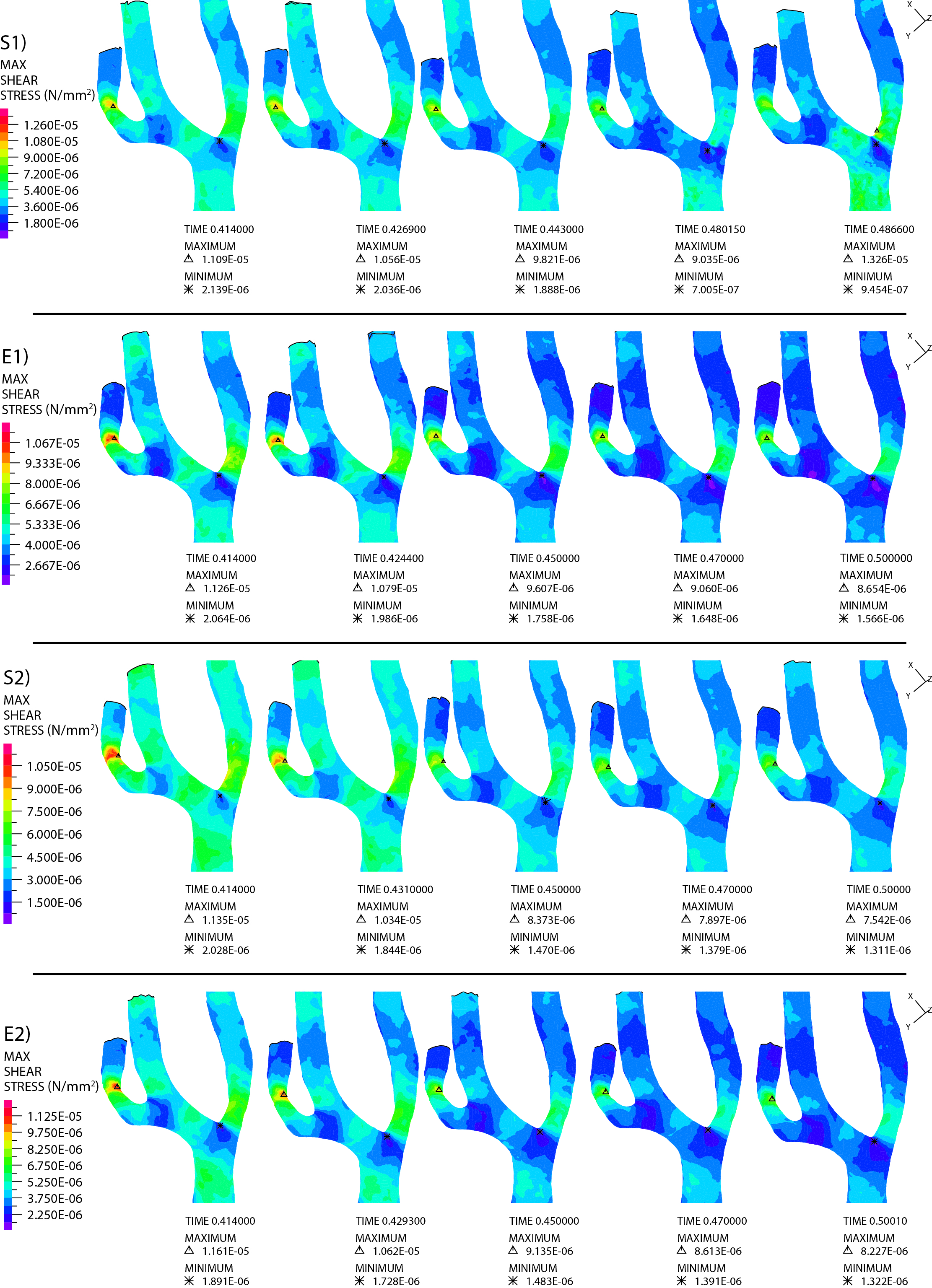


Fig. S 13. Shear stress on the third part of the MCA with the presence of emboli of different geometries. (The model of the embolus is specified in each row. All emboli released at the peak of systole.)

**References**

[1] Chueh JY, Wakhloo AK, Hendricks GH, Silva CF, Weaver JP, Gounis MJ. Mechanical characterization of thromboemboli in acute ischemic stroke and laboratory embolus analogs. American Journal of Neuroradiology 2011;32. https://doi.org/10.3174/ajnr.A2485.

[2] Song J, Zhu F, Qian Y, Ou C, Cai J, Zou X, et al. Morphological and hemodynamic differences between aneurysmal middle cerebral artery bifurcation and contralateral nonaneurysmal anatomy. Neurosurgery 2017;81. https://doi.org/10.1093/neuros/nyx093.
